# Synthesis, Properties and Antimicrobial Activity of 5-Trifluoromethyl-2-formylphenylboronic Acid

**DOI:** 10.3390/molecules25040799

**Published:** 2020-02-12

**Authors:** Agnieszka Adamczyk-Woźniak, Jan T. Gozdalik, Dorota Wieczorek, Izabela D. Madura, Ewa Kaczorowska, Ewa Brzezińska, Andrzej Sporzyński, Jacek Lipok

**Affiliations:** 1 Faculty of Chemistry, Warsaw University of Technology, ul. Noakowskiego 3, 00-664 Warsaw, Poland; jgozdalik@ch.pw.edu.pl (J.T.G.); izabela@ch.pw.edu.pl (I.D.M.); ewak@ch.pw.edu.pl (E.K.); ewabbrzezinska@gmail.com (E.B.); spor@ch.pw.edu.pl (A.S.); 2 Faculty of Chemistry, University of Opole, ul. Oleska 48, 45-042 Opole, Poland; dorwieczorek@uni.opole.pl (D.W.); jacek.lipok@uni.opole.pl (J.L.)

**Keywords:** acidity, antimicrobial, benzoxaborole, crystal, docking, equilibrium, formyl, Kerydin, phenylboronic, Tavaborole, trifluoromethyl

## Abstract

2-Formylphenylboronic acids display many interesting features, not only from synthetic but also from an application as well as structural points of view. 5-Trifluoromethyl-2-formyl phenylboronic acid has been synthesized and characterized in terms of its structure and properties. The presence of an electron-withdrawing substituent results in a considerable rise in the acidity in comparison with its analogues. In some solutions, the title compound isomerizes with formation of the corresponding 3-hydroxybenzoxaborole. Taking into account the probable mechanism of antifungal action of benzoxaboroles, which blocks the cytoplasmic leucyl-tRNA synthetase (LeuRS) of the microorganism, docking studies with the active site of the enzymes have been carried out. It showed possible binding of the cyclic isomer into the binding pocket of *Candida albicans* LeuRS, similar to that of the recently approved benzoxaborole antifungal drug (**AN2690**, Tavaborole, Kerydin). In case of *Escherichia coli* LeuRS, the opened isomer displays a much higher inhibition constant in comparison with the cyclic one. The antimicrobial activity of the title compound was also investigated in vitro, showing moderate action against *Candida albicans*. The compound reveals higher activity against *Aspergillus niger* as well as bacteria such as *Escherichia coli* and *Bacillus cereus*. In case of *Bacillus cereus*, the determined Minimum Inhibitory Concentration (MIC) value is lower than that of **AN2690** (Tavaborole). The results confirm potential of 2-formylphenylboronic acids as antibacterial agents and give a hint of their possible mechanism of action.

## 1. Introduction

**Scheme 1:**
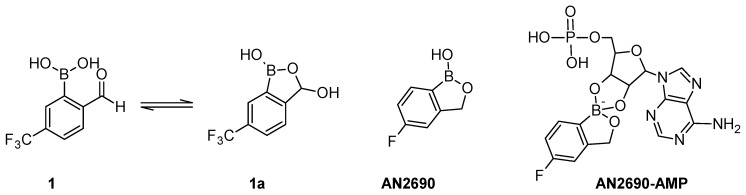
Structure of the 5-trifluoromethyl-2-formylphenylboronic acid (**1**), its cyclic isomer (**1a**), **AN2690** and the **AN2690-AMP** spiroboronate.

Boronic acids and especially arylboronic acids are of significant importance in chemistry. There are many examples of application of arylboronic acids, among others: building blocks in organic chemistry, sensors and receptors, polymers, active ingredients of drugs, functionalization of nanoparticles, boron neutron capture therapy (BNCT) and positron emission tomography (PET) [1,2,3,4,5]. Formyl-substituted phenylboronic acids additionally display high synthetic utility due to presence of a reactive aldehyde group. Reduction of the 2-formyl isomers is a highly versatile and straightforward method for benzoxaboroles’ synthesis [6]. The 2-formylphenylboronic acids are also very interesting from the structural point of view, since many interactions of the formyl group with the neighboring boronic unit are possible. Those interactions have been investigated by X-ray in the solid state [7]. One of the interesting phenomena of 2-formylphenylboronic acids is their possible isomerization in solution with the formation of cyclic isomers, which can be considered as 3-hydroxybenzoxaboroles [8]. As it was recently discovered, some 2-formylphenylboronic acids display antimicrobial activity, which was found to correlate with the amount of a cyclic isomer formed in DMSO (dimethylsulfoxide) solution [9]. At the same time, the presence of a fluorine substituent in arylboronic acids as well as in benzoxaboroles is crucial in their medicinal [10] and sensing applications. It enhances their acidity, resulting in binding the cis-diol bioanalytes at physiological conditions [11,12]. Fluorinated organic compounds show significantly different properties than analogous non-fluorinated ones. The former display higher lipophilicity and are able to form hydrogen bonds via the fluorine atom [13]. It is noteworthy that about 25% of known drugs contain the fluorine atom [14]. The recently approved benzoxaborole antifungal drug (**AN2690**, Scheme 1) also contains fluorine atom. The **AN2690** structure was the first representative of a novel benzoxaborole-based group of biologically active compounds with a unique mechanism of action based on blocking the LeuRS (Leucyl t-RNA synthetase) of microorganisms [6]. The molecular basis of AN2690 action as antifungal agent is formation of its spiroboronate with AMP (adenosine monophosphate, **AN2690-AMP**, Scheme 1).

The subject of the present work is the synthesis, structural study and spectral characterization as well as evaluation of the acidity and antimicrobial activity of 5-trifluoromethyl-2-formyl- phenylboronic acid (**1**, Scheme 1). The title compound is commercially available (CAS 1204580-94-8) and has been previously used synthetically [15], however not characterized either spectroscopically or in terms of its physicochemical properties. Compound **1** is able to tautomerize with formation of a cyclic isomer (**1a**), which resembles the structure of **AN2690** (Scheme 1)—the active ingredient of the antifungal drug Kerydin. Nothing is known however about the actual amount of **1a** in solution or antimicrobial activity of the title compound. All those issues are the subject of the current paper. Studies of isomers of **1**, including analog of **AN2690**, are in progress and will be reported in future.

## 2. Results and Discussion

### 2.1. Synthesis, Molecular and Crystal Structure

**Scheme 2:**
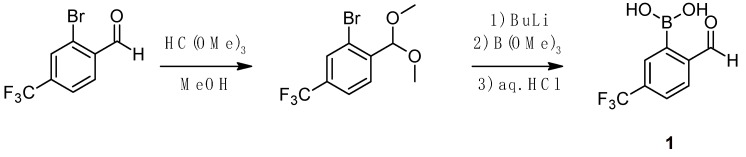
The synthesis of the 5-trifluoromethyl-2-formylphenylboronic acid (**1**).

The title compound was synthesized from the corresponding bromobenzaldehyde in a two-step reaction according to a typical procedure [10] (Scheme 2). The purity and homogeneity of the sample was confirmed by elemental analysis.

**Figure 1:**
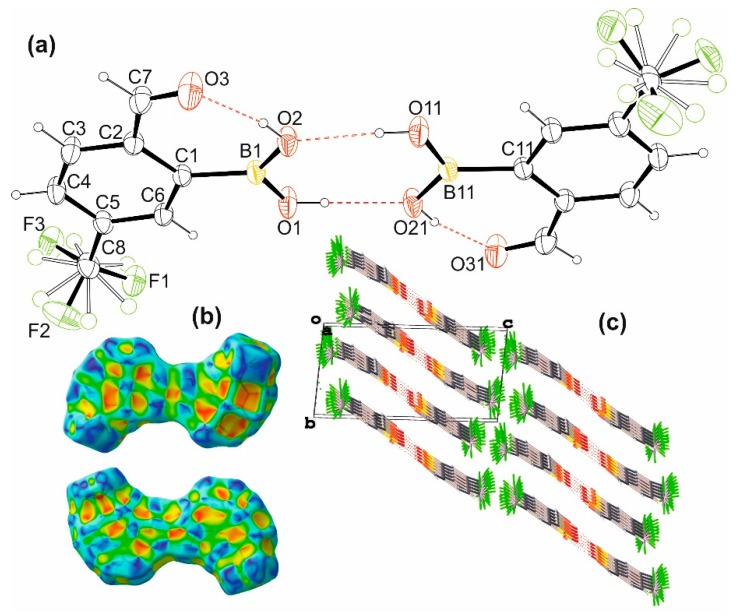
(**a**) Hydrogen bonded basic structural motif formed by two independent molecules **1-I** and **1-II**. Ellipsoids are given with 30% probability; (**b**) Molecular Hirshfeld surface (HS) with mapped surface index for the motif. Both sides of the HS surface are shown; (**c**) Packing diagram of **1**.

Molecules of **1** crystallize in the centrosymmetric space group of the triclinic system. Two crystallographically independent molecules (**1-I** and **1-II**) are present in an asymmetric part of the unit cell, forming a hydrogen bonded motif described at the second level of the Etter’s graph [16] as *R*^2^_2_(8), thus resembling the most popular synthon observed for boronic acids with the *syn*-*anti* conformation of the B(OH)_2_ moiety (Figure 1a). In the case of **1**, such a conformation is in a way forced by the presence of an intramolecular O-H∙∙∙O hydrogen bond with the formyl group present at *ortho* position. The geometry of all H-bonds observed in **1** are collected in Table 1 whereas other geometrical parameters, showing the likeness of both molecules (**1-I** and **1-II**), can be found in Table S1 and Table S2 in the Supplementary Materials. Both symmetrically independent molecules are essentially flat, excluding F atoms, with root-mean-square deviation from planarity (RMSD) of 0.025(1) and 0.077(1) Å for **1-I** and **1-II**, respectively. A close inspection with the aid of the Hirshfeld surface (HS) analysis [17] of intermolecular interactions between the basic structural motives revealed that stacking interactions are predominant in building a crystal (see corresponding red and blue areas on HS surface in Figure 1b). The shortest distances between planes containing the motif and related by the inversion and translation are 3.283(1) and 3.442(1) Å. Moreover, very weak C-H∙∙∙O hydrogen bonds between hydrogen atom of the formyl group and O1/O11 atom are also present, joining the motives into flat tapes running almost parallel to the (0–11) crystallographic plane. Taking into account the mentioned interactions a layer perpendicular to [001] direction and terminated with disordered CF_3_ groups is observed (Figure 1c). It is worth mentioning that two other structurally characterized *ortho* formyl substituted boronic acids, namely 2-formylphenyl- [7] and 2-formyl-3-fluorophenylboronic acid [8] are also flat and forming the *R*^2^_2_(8) basic structural motif as well as stacking columns.

### 2.2. Acidity Constant Determination

**Scheme 3:**
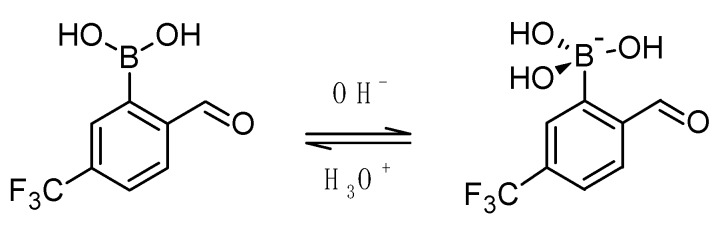
Acid–base equilibrium in 5-trifluoromethyl-2-formylphenylboronic acid (**1**).

**Figure 2:**
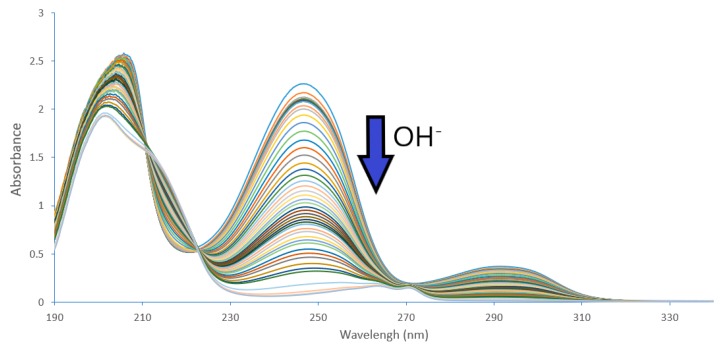
Observed change in spectrum during titration of compound **1** with sodium hydroxide.

The 5-trifluoromethyl-2-formylphenylboronic acid (**1**) similarly to other boronic acids is Lewis acid and exists in the acid–base equilibrium (Scheme 3). In alkaline conditions, boronic group assumes tetragonal form instead of a trigonal, flat form [18,19,20]. This causes change in UV spectrum which is shown in Figure 2. The p*K*_a_ of arylboronic acids is influenced by electronic as well as steric effects of the substituents. Introduction of the formyl group into position “2” of the boronic acids causes significant increase of acidity (drop in p*K*_a_). In case of aryl compounds with a fluorine substituent, both mesomeric and inductive effects take place, while the trifluoromethyl group displays only strong inductive effect [21]. The acidity of boronic trifluoromethyl derivatives is generally higher than that of analogous fluorine substituted molecules. It should be however noted that it also strongly depends on the position of the substituents (see Table 2 for values) [10,22,23,24].

The acidity constant of **1** was determined according to the previously reported method [25,26]. The obtained value (5.67 ± 0.01) is the lowest among analogous 2-formylphenylboronic acids and generally among other previously described substituted phenylboronic acids [3]. The determined value follows the general trend that replacing the F substituent with the CF_3_ substituent results in a drop of p*K*_a_ by approximately 1 unit (see Table 2 for values). A relatively high acidity of **1** should enhance diols binding, possibly facilitating formation of a spiroboronate with adenosine monophosphate, which may result in antimicrobial activity.

### 2.3. Spectral Characterization, Cyclization Equilibrium

**Scheme 4:**
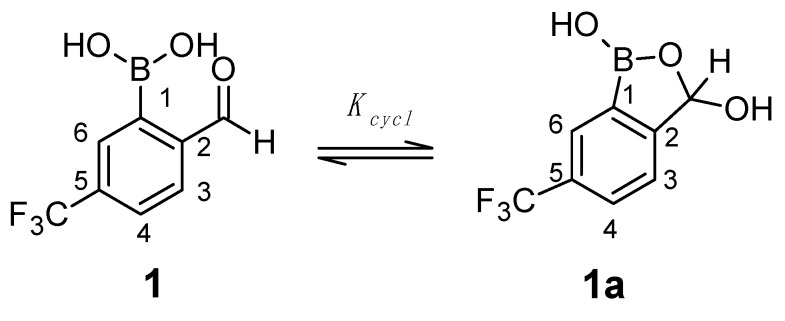
Atom numbering in **1** and **1a**.

2-Formyl-substituted boronic acids may exist in solutions in tautomeric equilibrium—open and cyclic form, which was previously described [8,9,10,27]. Until today, some halogen-substituted boronic acids were investigated [8] as well as 2,6-diformylphenylboronic acid [27] and all isomers of mono-fluoro-substituted 2-formylphenylboronic acids [9,10]. The ^1^H NMR (Nuclear Magnetic Resonance) spectra of compound **1** have been measured in several deuterated solvents (chloroform, benzene, DMSO, acetone and water) to investigate the influence of a solvent on possible tautomerization and formation of a cyclic isomer **1a** (Scheme 4). It was found that only isomer **1** was present in benzene and chloroform solutions (Table 3). Interestingly the proton of a formyl group in **1** couples with the H3 proton in CDCl_3_ solution, which has been confirmed on the basis of the ^1^H-^1^H COSY spectrum.

In contrast to chloroform and benzene solutions, certain amounts of **1a** contained the acetone, DMSO and aqueous solutions. In case of the title compound solutions in DMSO an interesting phenomenon was observed. In the event of low concentration of the sample (ca. 0.002M), a coupling between the hydrogen and hydroxyl group in position “3” of the oxaborole ring is manifested in the ^1^H NMR spectrum. The corresponding correlation peak was present in the ^1^H-^1^H COSY spectrum of the sample as well. However, during raise in concentration a simultaneous broadening of the signal corresponding to hydroxyl group is observed and the neighboring proton signal becomes a singlet. This can be attributed to formation of dimeric species with increasing concentration of the sample. However, the correlation signal is still present in the ^1^H-^1^H COSY spectrum of the most concentrated sample studied (0.02 M, see Supplementary Materials for picture). The **1**-**1a** equilibrium was investigated by ^1^H NMR and ^19^F NMR spectroscopy at 25 °C. The ratio of isomers was determined by integration of characteristic and well isolated signals corresponding to each of the isomers (Table 4). Four solutions differing by concentration have been prepared in DMSO (2.3 mg, 11.1 mg, 27.2 mg and 29.7 mg of **1** in 0.5 mL DMSO was dissolved) and four solutions in acetone (2.4 mg, 10.1 mg, 21.1 and 29.3 mg of **1** in 0.5 mL acetone was dissolved). At studied concentrations: (0.002–0.027M), the ratio of **1** and **1a** does not depend on the concentration of the sample. The values of cyclization constant (*K_cycl_*) in acetone, DMSO and D_2_O solutions were calculated according to the following Equation (1):

(1) $$K_{cycl}=\frac{I_{1a}}{I_{1}}$$

where *I_1a_* stands for the integrate of the signal corresponding to **1a** and *I_1_* stands for the integrate of the signal corresponding to **1** in the appropriate spectrum.

The ^13^C NMR chemical shifts of the title compound in CDCl_3_ are given in Table 5. The observed chemical shifts and coupling constant with fluorine atoms of the CF_3_ group and C5 carbon are typical and coherent with literature values [23,28]. Other signals in ^13^C were assigned on the basis of the ^1^H-^13^C HSQC (Heteronuclear Single Quantum Correlation) spectrum. A comparison with the literature values of the analogous fluorinated compound, namely 5-fluoro-2-formylphenylboronic acid, helped in assigning signals of carbon C2 of both forms [10]. In the carbon spectrum, no signals corresponding to trifluoromethyl group or C5, and C1 carbons of isomer **1a** were observed due to both specific shapes of the mentioned signals as well as low concentration of isomer **1a** in the sample.

### 2.4. Docking Studies of Interactions of AN2690 and 1/1a with Candida albicans’ and Eschericha coli LeuRS

**Figure 3:**
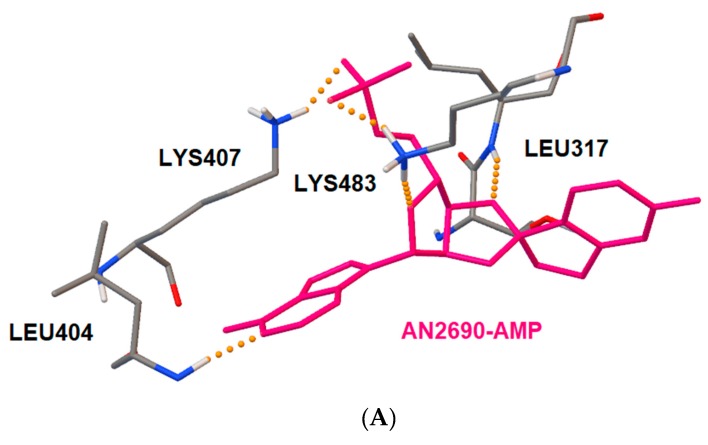

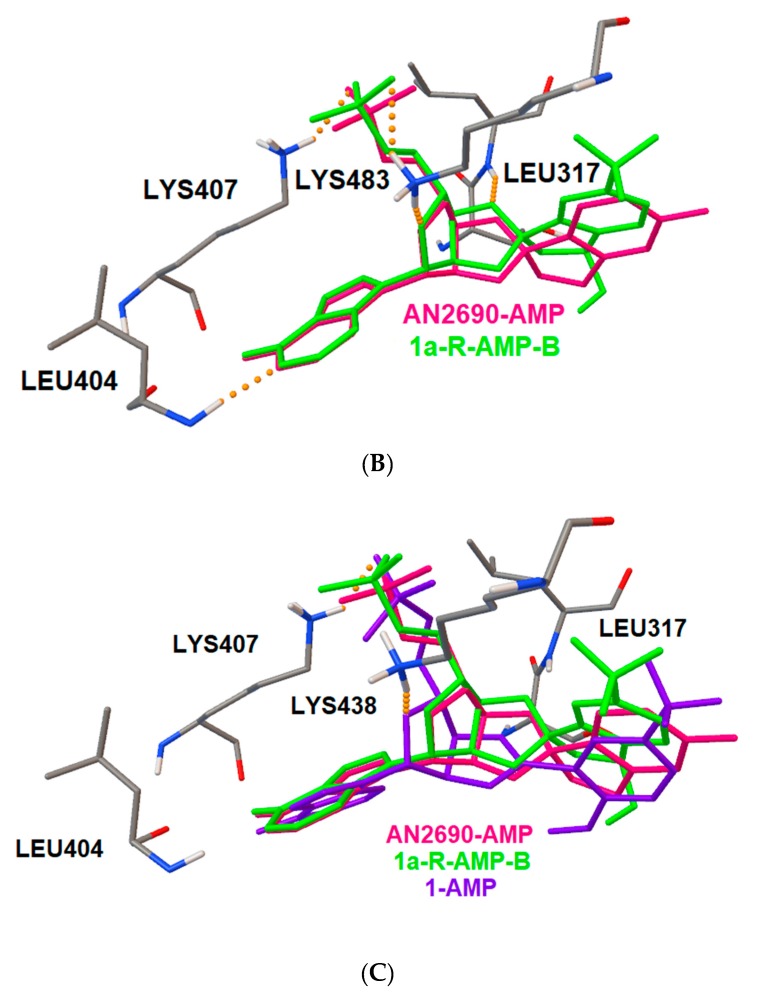
(**A**) The structure of **AN2690-AMP** spiroboronate docked into the *Candida albicans* LeuRS binding pocket (**B**) the **AN2690-AMP** spiroboronate (pink) along with **1a-AMP** spiroboronate (green) (**C**) **AN2690-AMP** spiroboronate (pink) along with **1a-AMP** spiroboronate (green) and **1-AMP** spiroboronate (purple) docked into *Candida albicans* LeuRS. Dotted lines stand for hydrogen bonds.

It is worth noting that a reasonable amount of the cyclic isomer **1a** is present in DMSO solution, which is used in the in vitro studies of antimicrobial properties of **1** (Table 4). In case of the previously reported isomeric fluoro-2-formylphenylboronic acids, the antimicrobial activity seemed to correlate with the amount of the cyclic isomer in DMSO solution [9]. It was concluded from structural similarities of the cyclic isomers to benzoxaborole drug **AN2690**. The molecular mechanism of action of **AN2690** has been established on the basis of structural studies of the crystal structure of the corresponding leucyl-RS synthetase—EC 6.1.1.4 [29,30]. It was found that **AN2690** forms a spiroboronate with adenosine monophosphate (AMP) within the editing domain of the cytoplasmic LeuRS of *C. albicans*. It is worth noting that LeuRS is also a possible molecular target in other microorganisms [6]. In order to prepare models for docking studies, crystal structures of the proteins with bound **AN2690** was taken from the Protein Databank and the ligand (the **AN2690-AMP** spiroboronate) has been removed from structures. To verify the docking procedure, the **AN2690-AMP** spiroboronate was docked into such an obtained protein resulting in structure analogous to the starting one (Figure 3a). With the docking procedure in hand, the binding the title compound **1** and its cyclic isomer **1a** was investigated to compare their potential in enzyme interactions with that of the **AN2690**. Needless to say that **1a** is a chiral molecule and each of the enantiomers can display different activity. Therefore, each of the enantiomers has been evaluated. In case of every input structure (see Supplementary Materials for structures) 300 docking procedures according to the genetic algorithm have been carried out. In case of all the possible spiroboronates, the starting position of the ligand was analogous to that of **AN2690** known from the experimental complex [29]. The search for binding sites covered the whole protein structure. Optimal structures of all the ligands and corresponding spiroboronates are given in Supplementary Materials. Figure 3b shows the **AN2690-AMP** spiroborate (pink) as well as analogous **1a-AMP** spiroboronate (green) docked into the *Candida albicans* LeuRS binding pocket. The position of all the spiroboronates (**AN2690-AMP**, **1-AMP** and **1a-R-AMP-B**) is similar (Figure 3c), which suggests that the mode of action of **1** against *Candida albicans* may be analogous to the known antifungal drug **AN2690**. 

To judge the binding of the investigated ligands, the binding energy was determined as a difference between the energy of a protein-ligand complex and the sum of the energy of ligand and a protein. Table 6 contains the lowest binding energy, number of structures in the best cluster along with mean binding energy and inhibition constants for the best cluster. The number of hydrogen bonds between ligand and the enzyme is also given. Supplementary Materials contain all the input and docked structures. The best binding energy of (−11.89 kcal/mol) displayed the **AN2690-AMP** spiroboronate forming five hydrogen bonds with the protein. The remaining ligands studied showed considerably higher energy of binding (weaker interaction). It is worth noting that the energies of binding along with the inhibition constants of **1a-AMP** spiroboronates are better than that of the **1-AMP** spiroboronate, which suggests that **1a** is the most probably the active isomer. However, only ligand **1a-R-AMP-B** forms as many hydrogen bonds as the parent drug ligand and displays similar inhibition constant (Table 6). It is worth to underline that due to chirality of molecule **1a** and probable differences in activity of optical isomers, the actual activity may be lower than it could be expected from amount of the **1a** in solution. The presented docking studies are in line with results of the in vitro studies of **1**/**1a** activity against *Candida albicans* described in Chapter 2.5. 

**Figure 4:**
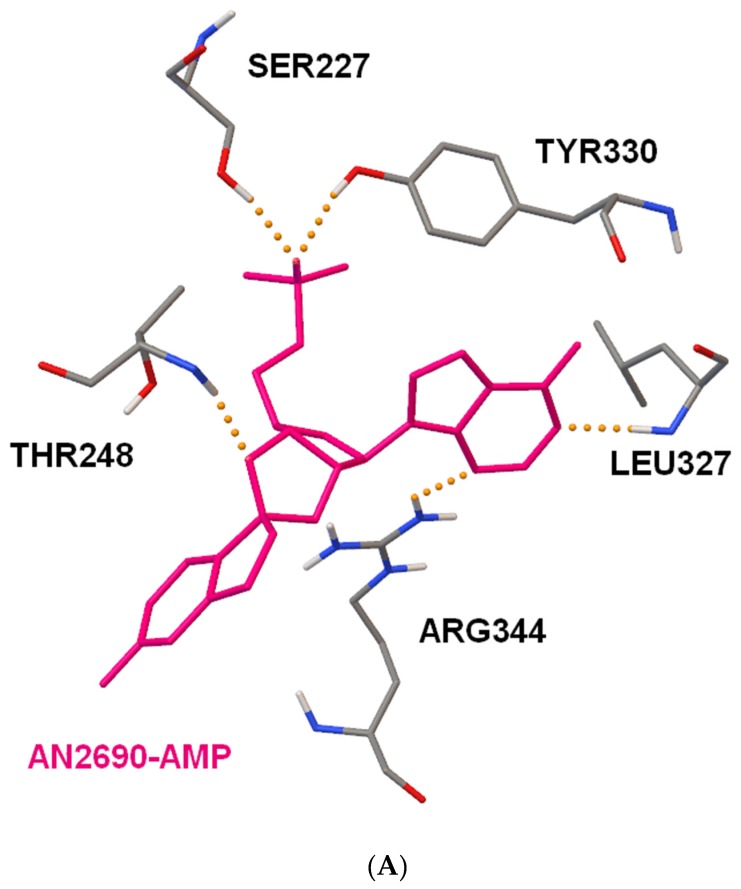

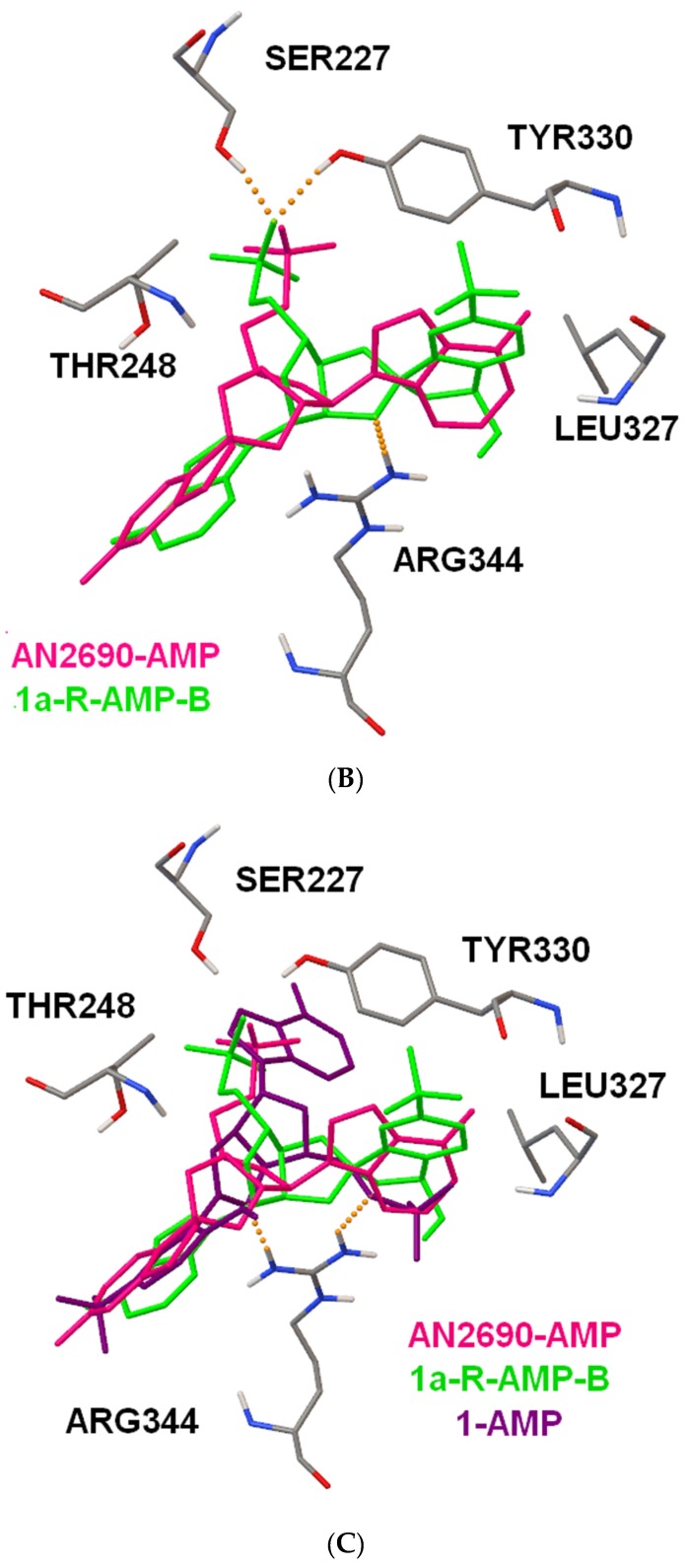
(**A**) The structure of **AN2690-AMP** spiroboronate docked into the *E. coli* LeuRS binding pocket (**B**) the **AN2690-AMP** spiroboronate (pink) along with **1a-AMP** spiroboronate (green) (**C**) **AN2690-AMP** spiroboronate (pink) along with **1a-AMP** spiroboronate (green) and **1-AMP** spiroboronate (purple) docked into *E. coli* LeuRS. Dotted lines stand for hydrogen bonds.

In the cases of the studied bacteria, there is no published crystal structure of LeuRS bound to **AN2690**. Therefore, the *E. coli* LeuRS (4ARI) complex with ligand containing no fluorine atom (**AN2679**) was taken from the protein databank [30] and used in docking studies. In this case, the docking procedure covered only the binding domain of the enzyme. The ligands studied were the same as in the case of *C. albicans* (Table 6). Interestingly, the docking studies show that in the case of *E. coli* the opened isomer spiroboronate (**1-AMP**) displays the highest inhibition constant (924.99 nM), comparable to that of **AN2690-AMP**, which is 865.19 nM. At the same time, all the closed isomers (**1a**) display inhibition constant at µM level (Table 6), which means that the opened isomer (**1**) should be more active in this case. It is worth to underline that the position of **1a-AMP** as well as **1-AMP** spiroboronates docked into the binding domain of *E. coli* LeuRS is different than that of the corresponding **AN2690-AMP** spiroboronate. It might suggest a different mechanism of action of **1** and **1a** on bacterial strains. The structures docked into the enzyme have been presented at Figure 4.

### 2.5. In Vitro Studies of Antimicrobial Activity of **1**

**Figure 5:**
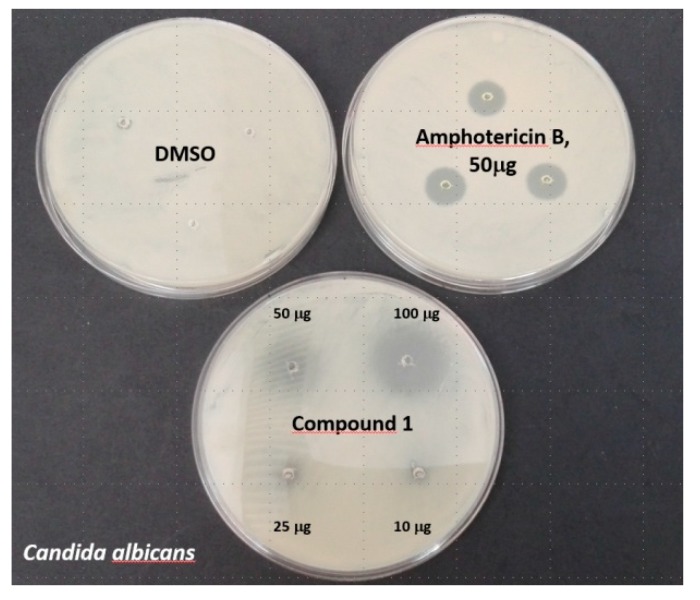
Results of agar diffusion method of activity against *Candida albicans* for a negative probe (DMSO), positive probe (50 µg of amphotericin B) and compound **1**.

Antimicrobial activity of **1** was established using both the agar diffusion method and by determination of minimal inhibitory concentration (MIC) values. DMSO has been used as a negative probe while the **AN2690** and appropriate drugs have been treated as positive controls (Figure 5). The results of both methods are consistent, showing moderate antifungal as well antibacterial activity of 2-formyl-5-trifluoromethyl phenylboronic acid (**1**).

Gram (+) and Gram (−) bacterial strains were chosen as the representatives of two, different regarding the structure of the cell wall microbiota of various susceptibility towards the action of antibiotics. Whereas tested fungi were chosen because of their belonging to unicellular (*C. albicans*) and multicellular (*A. niger*) pathogenic fungi. In most of the studied cases, the title compound (**1**) limits the growth of the microorganisms. However, only at the highest dose studied (100 µg) it totally inhibits the growth of *Candida albicans* and *Aspergillus niger* with a relatively small zone of inhibition of 8 and 5 mm, respectively (Table 7). 

The MIC values are also moderate, especially for *Candida albicans* (250 µg/mL, Table 8). The determined values of the diameter of the zone of inhibited growth of *Candida albicans* and *Aspergillus niger* show that they are several times higher for **AN2690** than amphotericin B. It may result from a high diffusion of **AN2690** within the applied media. Taking into account the MIC values, **1** is more active against *Escherichia coli* (125 µg/mL) and *Aspergillus niger* (32 µg/mL) than against *Candida albicans* (250 µg/mL). The reason for the lower activity of **1** towards tested fungi may be launched to low concentration of the presumably active isomer (**1a-R**) in water, which is the main component of the media applied for MIC determination. Docking studies show that in the case of *E. coli*, it is isomer **1** that displays three orders of magnitude higher inhibition constant than both **AN2690** and isomer **1a**. This is in line with a relatively high observed antibacterial activity of **1**, which is a predominant form in aqueous solution. Needless to say that the issues of cell membrane permeability of the microorganism must be also taken into account. The lowest MIC value (highest activity) was determined for *Bacillus cereus* (8 µg/mL). It is significantly lower than that of the **AN2690** (62.5 µg/mL) but still higher than that of the reference drug (streptomycin, 4 µg/mL). The observed phenomenon may be addressed to a different structure of *B. cereus* membrane, as it is the representative of Gram positive bacteria, which are generally more sensitive for the action of antibiotics [31]. Worth noticing is a relatively high MIC value of **AN2690** for *Bacillus cereus*, which is in line with previous findings on a rather low interactions of **AN2690** with bacterial LeuRS [32].

## 3. Materials and Methods

### 3.1. Synthesis and Isolation

2-Bromo-4-trifluoromethylbenzaldehyde (9.82 g, 38.7 mmol) was dissolved in 16 mL of methanol. Concentrated H_2_SO_4_ (0.2 mL) was added and 5.33 g (50.3 mmol) of trimethyl orthoformate was added dropwise. The solution was refluxed for 1 h at 72 °C in an oil bath and left to cool down. Then the solution was brought to pH 11 with a concentrated solution of NaOMe in methanol (NaOMe was synthetized by adding sodium metal into anhydrous methanol). After distillation of the volatiles at reduced pressure (35–40 °C), the product was distilled under vacuum (140–200 °C) to give 8.77 g, 29.4 mmol of 1-bromo-2-(dimethoxymethyl)-5-trifluoromethylbenzene as a colorless liquid (yield 75%). ^1^H NMR (300 MHz, Chloroform-d), δ/ppm: 7.83 (m, 1H), 7.74 (m, 1H), 7.60 (m, 1H) 5.57 (s, 1H), 3.39 (s, 6H); ^19^F NMR (282 MHz, Chloroform-d), δ/ppm: −80.94 ppm (s, 3F).

62 mL of dry Et_2_O and 12 mL THF under argon flow were cooled down to −75 °C using dry ice/acetone bath. 2.5M *n*-butyllithium in hexanes (12.0 mL, 31.43 mmol) was added. Next, 1-bromo-2-(dimethoxymethyl)-5-trifluoromethylbenzene (8.71 g, 29.16 mmol) was added dropwise while keeping the temperature under −70 °C. The colour of the mixture changed into green; the solution was stirred for 1 h. Then 3.4 g (34.0 mmol) of triethyl borate was added slowly, keeping the temperature under −70 °C; the colour of the mixture turned into greenish yellow; the solution was stirred for 1 h. Cooling bath was removed and the solution was brought to pH 3 with 3M aq. HCl, while the temperature rose to 5 °C. The aqueous layer was separated and extracted with Et_2_O (2 × 50 mL). The organic layers were combined and the solvent was partially removed under vacuum. After precipitation of a white solid 50 mL of water was added and distillation was continued to remove volatiles. The remaining solution was left for several hours in the refrigerator (4 °C), then two phases was observed: aqueous and oil. The aqueous phase was left for crystallization. After water evaporation, white crystals of pure title compound were received (0.0563 g). To the oil phase during stirring hexane was added resulting in separation of a solid. The white precipitate was filtered off. Pure product **1** was obtained from the filtrate by crystallization from an acetone:aqueous solution (1.59 g). The total amount obtained was 1.64 g (26% yield). The purity of product was confirmed on the basis of elemental analysis: calculated for: C_8_H_6_BF_3_O_3_; C: 44.09, H: 2.77, found: C: 44.06 ± 0.03, H: 2.75 ± 0.04. ^1^H NMR (ppm): 10.04 (m, CHO), 8.54 (m, 1H), 8.06 (m, 1H), 7.97 (m, 1H), 7.13 (bs, OH); ^11^B NMR (ppm): 27; ^19^F NMR (ppm): 64.4. Spectra of **1** in various solvents are given in the text, Table 3 and Table 5 as well as in the Supplementary Materials.

### 3.2. Single Crystal X-Ray Diffraction 

Single crystals of **1** suitable for X-ray diffraction experiment were obtained by crystallization from water. Data were collected at room temperature on a Gemini A Ultra Diffractometer (Rigaku Oxford Diffraction, Wroclaw, Poland) with graphite monochromated Mo/Ka radiation (λ = 0.71073 Å). Data collection and data reduction were performed in the CrysAlis^Pro^ program [33]. To solve and refine the structure, OLEX-2 version 1.2 [34] with incorporated SHELXT [35] and SHELXL (the full-matrix least-squares technique) programs [36,37] were used. All non-hydrogen atoms were refined with anisotropic temperature factors. The OH-groups of hydrogen atoms were refined freely with fixed O-H distance to 0.85(1) Å. The remaining H-atoms were placed in calculated positions with fixed isotropic thermal parameters (U_iso_(H) = 1.2 × [U_eq_(C)]). In both symmetrically independent molecules (**1-I** and **1-II**) the -CF_3_ group showed rotational disorder. The final model with enhanced rigid body restrains (RIGU) and fixed C-F distances to 1.320(2) Å contains refined three distinct position of each group with the site occupation factor of 0.464(12), 0.352(14), 0.184(12) and 0.464(12), 0.359(14), 0.176(12) for **1-I** and **1-II** molecule, respectively. Molecular and packing diagrams were generated using ORTEP-3 for Windows (Oak Ridge National Lab, Oak Ridge, USA) [37] and OLEX-2 [34] programs, respectively. Crystal data for **1**: C_8_H_6_BF_3_O_3_ (M = 217.94 g/mol): triclinic, space group P-1 (no. 2), a = 7.6512(3) Å, b = 7.6813(4) Å, c = 15.4975(6) Å, α = 94.121(4)°, β = 98.795(3)°, γ = 91.504(4)°, V = 897.13(7) Å^3^, Z = 4, T = 298 K, μ(MoKα) = 0.158 mm^−1^, D_calc_ = 1.614 g/cm^3^, 19471 reflections measured (6.974° ≤ 20 ≤ 65.7°), 6111 unique (R_int_ = 0.0308, R_sigma_ = 0.0310), which were used in all calculations. The final R_1_ was 0.0513 (I > 2σ(I)) and wR2 was 0.1586 (all data). CCDC (Cambridge Crystallographic Data Centre) 1959978 contains the supplementary crystallographic data for this paper. These data can be obtained free of charge from the CCDC, 12 Union Road, Cambridge CB2 1EZ, UK; Fax: +44 1223 336033; E-mail: deposit@ccdc.cam.ac.uk.

### 3.3. Acidity Constant Determination

The acidity constant was determined by spectrophotometric method according to the previously reported description [22,25,26]. The title compound was dissolved in aqueous phosphate buffer with pH = 6.42 and acidified to pH = 2 by hydrochloric acid. Next, the solution was titrated by 0.25 M natrium hydroxide to pH = 8; pH value was measured by a standard combined glass electrode with temperature control, performed on the pH-meter (Hanna Edge, Woonsocked, RI, USA). Electrode was calibrated on the following buffer solutions: 4.01, 7.01, 9.18 and 10.01 (Hanna Instruments, Woonsocked, RI, USA). Spectrophotometric scan from 190 nm to 400 nm was done after each drop of titrant in pH range between 3.5 and 6.5, performed on the UV-3100 PC Spectrophotometer (VWR, Leuven, Belgium). Outside of the above-mentioned range, scans were done every change of the 0.5 pH value. Further technical details are presented in Supplementary Materials.

### 3.4. NMR Measurements

All NMR experiments (^1^H, ^11^B, ^13^C, ^19^F NMR, ^1^H-^1^H COSY and ^1^H-^13^C HSQC) were performed on Bruker Avance 300 MHz (BioSpin, Poznan, Poland). For hydrogen and carbon spectrum residual signals from solvents were used as reference signals. For boron spectrum—BF_3_·Et_2_O and for fluorine spectrum—CCl_3_F were used as external references.

### 3.5. Docking Studies

The structures of a leucyl-RS synthetase from *Candida albicans* as well as *Escherichia coli* have been taken from the Protein Databank [38]. The protein structure has been processed by AutoDock Tools [39] to remove water molecules, connect hydrogen atoms and calculate partial charges. The charges were calculated on the basis of standard force field parameters applied in AutoDock 4.2. Since the standard parameters in AutoDock do not cover boron atom, the following force-field parameters have been introduced: Rii (sum of vdW radii of two like atoms in Å) = 1.98; epsii (vdW well depth in kcal/mol) = 0.034; vol (atomic solvation volume in Å^3^) = 4.6000; solpar (atomic solvation parameter) = −0.00110; Rij_hb (H-bond radius of the heteroatom in contact with a hydrogen in Å) = 0.0; epsij_hb (well depth of H-bond in kcal/mol) = 0; H-bond (integer indicating type of H-bonding atom (0 = no H-bond) = 0; rec index (initialized to −1, but later on holds count of how many of this atom type are in receptor) = −1; bond index were used in AutoDock to detect bonds; see "mdist.h", enum {C,N,O,H,XX,P,S} = 4. The structures of investigated molecules (**1**, **1a**) have been generated in Avogadro and optimized in Gaussian 03 (b3lyp, 6–311g (2d,p) opt freq.) (Gaussian Inc., Wallingford, CT, USA). In case of molecule **1**, the position of the atoms has been taken from the crystal structure. The structures of spiroboronates with AMP were prepared by connection of optimized molecules with the AMP molecule taken from crystal structure of the **AN2690-AMP** spiroboronate found in the crystal phase [29]. The structures of such obtained spiroboronates were not optimized.

### 3.6. Microbial Activity

Our investigations addressed to microbial activity of 5-trifluoromethyl-2-formylphenylboronic acid, were performed towards four representatives belonging to different taxa of microorganisms: filamentous fungi—*Aspergillus niger* LOCK 0440, yeast—*Candida albicans* LOCK 001, Gram-positive bacteria—*Bacillus cereus* CCM 2010 and Gram-negative bacteria—*Escherichia coli* CCM 5172. Fungal species used for those experiments were obtained from the Institute of Fermentation Technology and Microbiology (University of Technology, Łódź, Poland), whereas examined bacteria were purchased from Czech Collection of Microorganisms (Masaryk University, Prague, Czech Republic). Before the examinations, *A. niger* was routinely cultured in potato dextrose agar at 27 °C, *C. albicans* in yeast extract-peptone-dextrose (YPD) medium at 27 °C while bacteria were pre-grown in nutrient broth at 34 °C.

Antimicrobial activity of tested compound was established using both the agar-diffusion method and by determining MIC values. For the diffusion tests, 0.5 mL of appropriate inoculum containing 10^6^–10^7^ spores or bacterial cells was placed on the surface of the solidified media suitable for the growth of fungi or bacteria respectively and allowed to dry. The *A. niger* spore suspension was prepared by washing the surface of the 10- to 14-day-old cultures with 0.05% Tween solution and quantified using the Thom’s chamber. In case of the rest of microorganisms the 24-h old cultures, diluted in a proper medium if necessary, were used. In such prepared cultures, 2-mm wells were cut and filled with 15 μL DMSO containing 100, 50, 25 or 10 μg of the tested compound. As the controls DMSO, 50 µg of **AN2690** and solutions of 50 μg of the appropriate antibiotic were used. Cultures of *Aspergillus niger* and *Candida albicans* were incubated for 48 h, while the cultures of other examined strains for 24 h. Experiments were carried out at least in triplicate. Antimicrobial activity was evaluated based on the comparison of the diameter of the zone of inhibition of microbial growth around the wells, measured both in controls and experimental treatments.

The Minimal Inhibitory Concentration (MIC) was determined by a serial dilution method. For this purpose, 2 mL of microbial cultures containing 10^6^ CFU per 1 mL of medium were prepared. Media used in MIC evaluation were Czapek Medium for *A. niger*, YPD medium for *C. albicans* and nutrient broth for *E. coli* and *B. cereus.* Stock solutions of **1** and **AN2690** were prepared by dissolving in DMSO. To obtain the set of experimental concentrations, the stock solution was serially diluted and placed to cultures, ensuring that the final concentration ranged from 1 to 500.0 µg/mL. Depending on the species of microorganism, appropriate cultures were incubated for 72 h (*A. niger*), 48 h (*C. albicans*) at 25 °C or 24 h at 34 °C (*E. coli*, *B. cereus*). The experiments were performed in triplicate. The MIC values were determined based on the lowest concentration of tested compound required to visual inhibition of fungal or bacterial growth.

## 4. Conclusions

The 2-formylphenylboronic acid containing the trifluoromethyl substituent at *meta* position (**1**) has been synthesized and characterized in terms of its structure and properties. The molecule displays typical dimeric structural motive in the solid state and a relatively high acidity. In DMSO, acetone and aqueous solutions **1** isomerizes with the formation of a cyclic isomer (**1a**) resembling the benzoxaborole structure. Antimicrobial activity of **1** and **AN2690** against *Candida albicans*, *Aspergillus niger* as well as *Escherichia coli* and *Bacillus cereus* has been evaluated. Docking studies of an antifungal benzoxaborole drug **AN2690** (Tavaborole, Kerydin), **1** and **1a** showed possible binding of the cyclic isomer **1a** into the binding pocket of *Candida albicans*’ cytoplasmic LeuRS. On the contrary, in case of the bacterial LeuRS (*E. coli*), isomer **1** displays higher inhibition constant than both **1a** and **AN2690**. The antimicrobial activity of the title compound investigated in vitro is in line with those findings, showing moderate action against *Candida albicans*, which may result from relatively low amount of **1a** in aqueous media. The title compound reveals higher activity against bacteria such as *Escherichia coli* and *Bacillus cereus*. In case of the latter, the determined MIC value is lower than that of **AN2690**. The considerable antibacterial activity of **1** can result from both its interactions with bacterial LeuRS (higher inhibition constant in comparison with **AN2690** and **1a**) as well as high concentration of **1** isomer in aqueous media. The results confirm a potential of 2-formylphenylboronic acids as antibacterial agents and give a hint of their possible mechanism of action.

## Figures and Tables

**Table 1:** Geometry of hydrogen bonds in **1**.

	H∙∙∙A	D∙∙∙A	D-H∙∙∙A
*Intramolecular*			
O2‒H2…O3	1.82(1)	2.619(1)	164(2)
O21‒H21…O31	1.81(1)	2.605(1)	164(2)
*Intermolecular*			
O1‒H1…O21	1.95(1)	2.774(1)	177(2)
O11‒H11…O2	1.97(1)	2.779(1)	174(2)
C7‒H7…O1 ^i^	2.61	3.254(2)	127
C71‒H71…O11 ^ii^	2.56	3.220(2)	128

Symmetry operations: (i) −1 + *x*,*y*,*z*; (ii) 1 + *x*,*y*,*z*.

**Table 2:** Selected boronic acids and their p*K*_a_ values.

General Formula	Substituent	p*K*_a_ Value	Reference
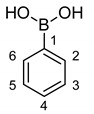	-	8.86	[10]
	3-F	7.50 ± 0.02	[22]
	3-CF_3_	7.85 ± 0.05	[23]
	2-CHO	7.31	[24]
	2-CHO, 5-F	6.72 ± 0.03	[10]
	2-CHO, 5-CF_3_ (**1**)	5.67 ± 0.01	This work

**Table 3:** ^1^H,^11^B and ^19^F NMR chemical shifts of the title compound in various solvents.

	^1^H NMR						^11^B	^19^F
Solvent (Form)	B(OH)_2_	CHO		H3	H4	H6		
		OH	H					
CDCl_3_ (**1**)	7.13 (s)	10.04 (m)		8.54 (m)	7.97 (m)	8.06 (d) ^3^*J*_H5_ = 7.8	27 (s)	−63.44
C_6_D_6_ (**1**)	7.04 (s)	9.04 (s)		8.55 (m)	7.20 (m)	6.73 (d) ^3^*J*_H5_ = 7.8	27 (s)	−63.29
(CD_3_)_2_CO (**1**)	7.84 (s)	10.40 (s)		8.10 (m)	7.93 (m)	8.15 (m)	29 (s)	−62.94
(CD_3_)_2_CO (**1a**)	8.50 (s)	6.38 (d) ^3^*J*_H_ = 7.9	6.22 (d) ^3^*J*_OH_ = 8.1	8.01 (m)	7.85 (m)	7.70 (m)	31 (s)	−61.98
(CD_3_)_2_SO (**1**)	8.47 (s)	10.24 (s)		7.89 (m)	7.91 (m)	8.07 (m)	30 (s)	−61.67
(CD_3_)_2_SO (**1a**)	9.57 (s)	7.23 (d) ^3^*J*_H_ = 8.1	6.25 (d) ^3^*J*_OH_ = 8.1	8.01 (m)	7.85 (m)	7.64 (m)	30 (s)	−60.73

Abbreviations: s (singlet), d (dublet), m (multiplet), *J* (coupling constant given in Hertzs (Hz)).

**Table 4:** Cyclization constant of title compound in various solvents (based on ^1^H NMR).

Solvent	Cyclization Constant	^1^H/^19^F NMR Shift of the Cyclic Form (1a)	^1^H/^19^F NMR Shift of The Opened Form (1)
(CD_3_)_2_SO	0.59 ± 0.01	6.25/−60.73	10.24/−61.67
(CD_3_)_2_CO	0.24 ± 0.02	6.22/−61.98	10.40/−62.94
D_2_O	0.26 ± 0.03 *	6.36/−62.22	10.06/−63.26

* Due to very low solubility of **1** in D_2_O the spectrum is of a relatively low signal to noise ratio.

**Table 5:** ^13^C NMR chemical shifts of the title compound (75 MHz, DMSO-d_6_).

Form	CHO/CH-OH	CF_3_	C1	C2	C3	C4	C5	C6
**1**	193.82 (s)	124.5 (q) ^1^*J*_F_ = 270.5	140.8 (bs)	142.1 (d) ^4^*J*_F_ = 1.1	129.5 (m)	125.7 (m)	132.2 (q) ^2^*J*_F_ = 31.5	129.9 (s)
**1a**	97.06 (s)	Not Observed	Not Observed	158.9 (s)	126.8 (q) ^3^*J*_F_ = 3.7	127.8 (m)	Not Observed	123.8 (s)

The abbreviations: s (singlet), b (broad), m (multiplet), q (quartet), J (coupling constant given in Hertzs (Hz)).

**Table 6:** Chosen results of docking studies of spiroboronates of **AN2690**, **1** and **1a** with LeuRS of *Candida albicans* and *Escherichia coli.*

	Ligand	The Lowest Binding Energy [kcal/mol]	Number of Structures	Mean Binding Energy [kcal/mol]	Inhibition Constant	Number of Hydrogen Bonds
*Candida albicans*	**AN2690-AMP**	−11.98	17	−10.80	1.93 nM	5
	**1a-R-AMP-A**	−10.77	2	−10.21	12.77 nM	4
	**1a-R-AMP-B**	−11.83	3	−11.66	2.12 nM	5
	**1a-S-AMP-A**	−10.75	19	−10.42	13.08 nM	3
	**1a-S-AMP-B**	−11.59	4	−10.94	3.17 nM	4
	**1-AMP**	−11.05	2	−10.77	7.95 nM	2
*Escherichia coli*	**AN2690-AMP**	−8.27	22	−7.55	865.19 nM	5
	**1a-R-AMP-A**	−7.54	21	−6.90	2.99 μM	2
	**1a-R-AMP-B**	−8.15	2	−7.81	1.09 μM	3
	**1a-S-AMP-A**	−7.54	12	−6.98	2.96 μM	2
	**1a-S-AMP-B**	−7.72	4	−7.45	2.21 μM	3
	**1-AMP**	−8.23	4	−7.75	924.99 nM	2

**Table 7:** The diameter of the zone of inhibited growth of the microorganisms in millimeters.

	10 µg	25 µg	50 µg	100 µg	AN2690 (50 µg)	Antibiotic (50 µg)
*Candida albicans*	0	9	13 ± 2	17 ± 3 (8 ± 1)	(53)	(10 ± 1) *
*Aspergillus niger*	0	8 ± 1	13 ± 2	26 ± 4 (5 ± 1)	(62)	(9 ± 1) *
*Escherichia coli*	2 ± 1	5 ± 1	7 ± 1	9 ± 1	(22)	(16 ± 1) ^#^
*Bacillus cereus*	12	15 ± 1	18	19 ± 1	(14)	(21 ± 1) ^#^

* Amphotericin B, ^#^ Streptomycin. Values in parentheses () mean total inhibition of the growth of the microorganism.

**Table 8:** Minimal inhibitory concentrations (MIC) of **1**, **AN2690** and standard drugs (amphotericin and streptomycin).

	MIC [µg/mL]		
	1	AN 2690	Antibiotic
*Candida albicans*	250	2	≤ 1 *
*Aspergillus niger*	32	≤ 1	-
*Escherichia coli*	125	7.8	2 ^#^
*Bacillus cereus*	8	62.5	4 ^#^
	* Amphotericin B, ^#^ Streptomycin		

## Supplementary Materials

The following are available online at https://www.mdpi.com/1420-3049/25/4/799/s1: Figures S1–24: NMR spectra of **1** in various solvents, S25–44: Input and optimal structures in docking studies. Figures S45–52: Examples of results of agar diffusion method. Tables S1 and S2: Bond lengths and selected bond angles in molecules of 1—data from X-ray studies. Table S3: Values of acidity constant determined in triplicate. Tables S4–23: Coordinates of the input and optimal structures in docking studies.
