# Supplementary material for: Synthesis, Properties and Antimicrobial Activity of 5-Trifluoromethyl-2-formylphenylboronic Acid
Source: Molecules. 2020 Feb 12;25(4):799. doi: 10.3390/molecules25040799 (PMC7070739; doi:10.3390/molecules25040799)
Supplement: Supplementary file 1 [file molecules-25-00799-s001.pdf]

Supplementary data of manuscript entitled:

Synthesis, properties and antimicrobial activity of 5-trifluoromethyl-2-formylphenylboronic acid

Authors: Agnieszka Adamczyk-Woźniak* [1], Jan T. Gozdalik[1], Dorota Wieczorek [2], Izabela D. Madura [1], Ewa Kaczorowska [1], Ewa Brzezińska [1], Andrzej Sporzyński[1], Jacek Lipok [2]

[1] Faculty of Chemistry, Warsaw University of Technology, ul. Noakowskiego 3, 00-664 Warsaw, Poland;
[2] Faculty of Chemistry, University of Opole, ul. Oleska 48, 45-042 Opole, Poland;
* Correspondence: agnieszka@ch.pw.edu.pl;

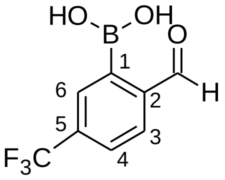

*Fig. S1. Title compound (**1**) with atom numbering scheme.*

Table of contents

Table of contents 1
1. NMR spectra of **1** in $CDCl_3$ 2
2. NMR spectra of **1** in C_6D_6 5
3. NMR spectra of **1/1a** in DMSO-d_6 7
4. NMR spectra of **1/1a** in acetone-d_6 12
5. NMR spectra of **1** in D_2O 13
6. Selected bonds lengths and torsional angles in **1**. 15
7. Acidity constant determination 16
8. Docking studies – input and optimal structures 17
9. Pictures of chosen results of diffusion agar method 37

1. NMR spectra of **1** in $CDCl_3$

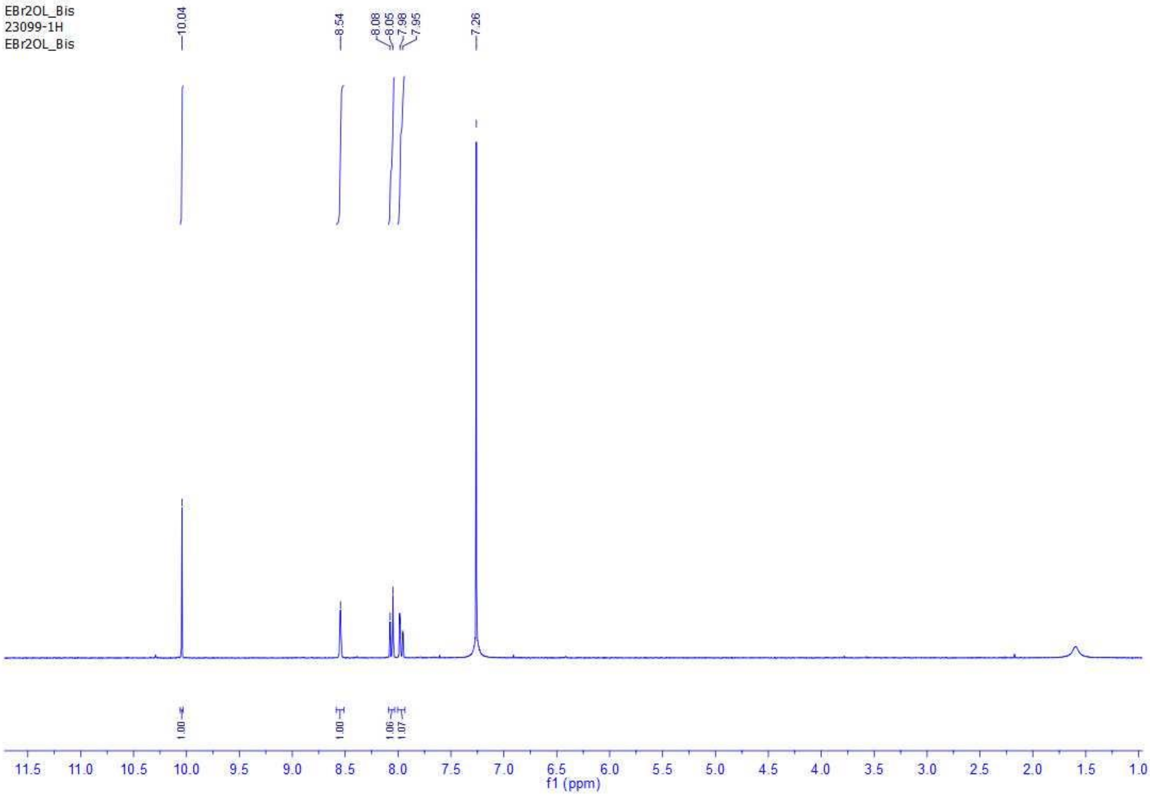

*Fig. S2. ^{1}H NMR spectrum of **1** in $CDCl_3$*

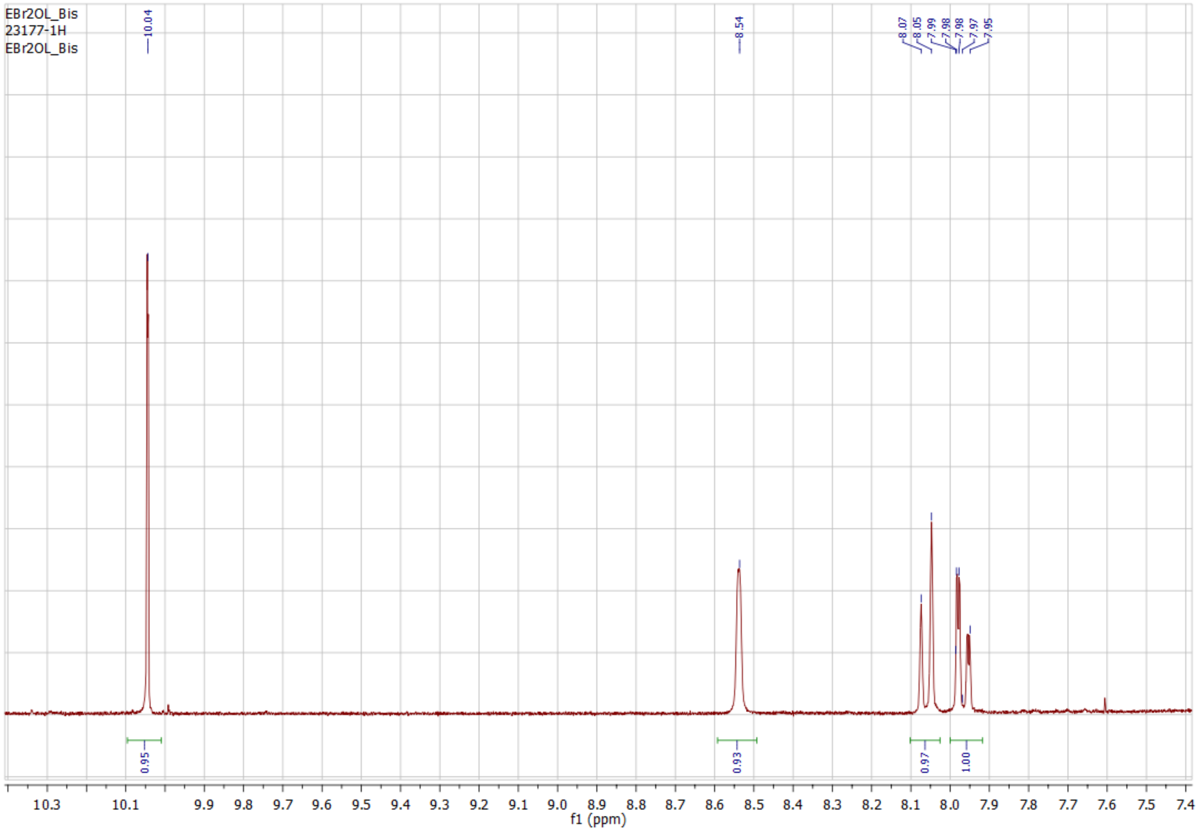

*Fig. S3. ^{1}H NMR spectrum of **1** in $CDCl_3$ (expanded).*

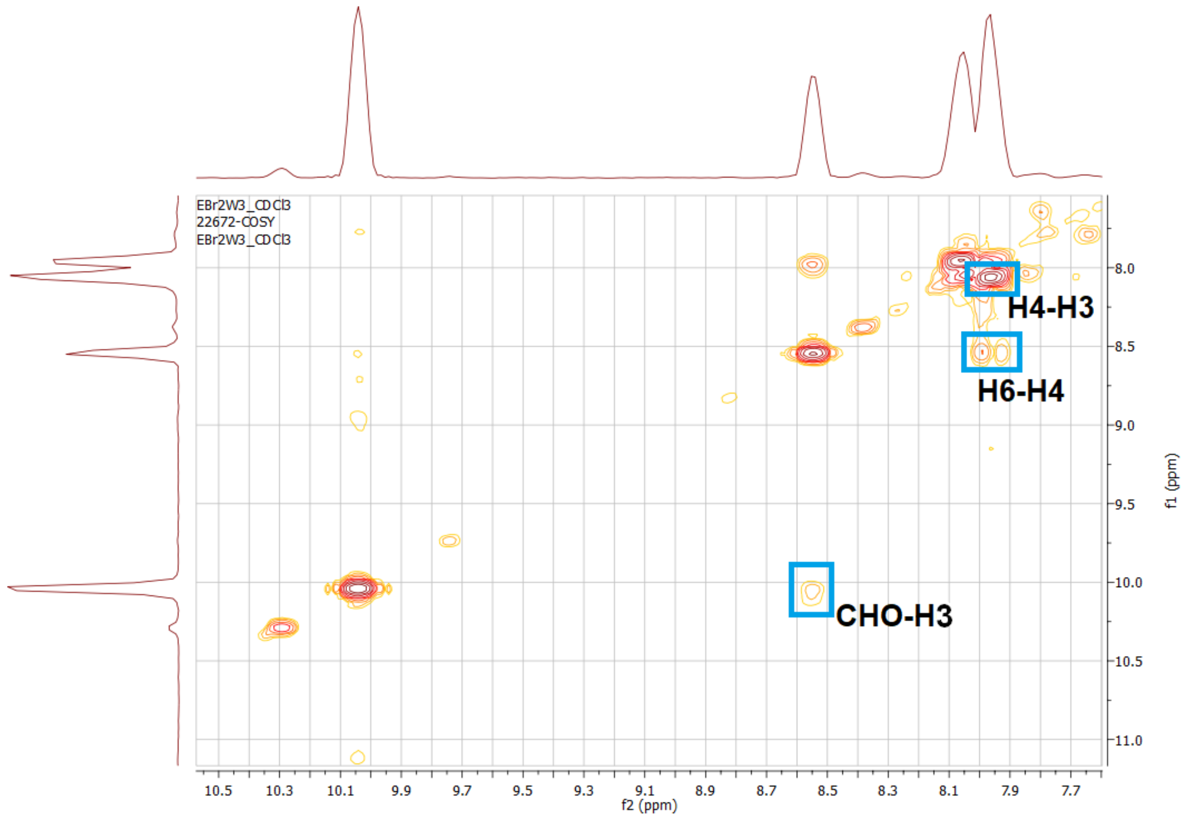

Fig.S4. [1]*H,*[1]*H COSY of* ***1*** *in CDCl*$_3$

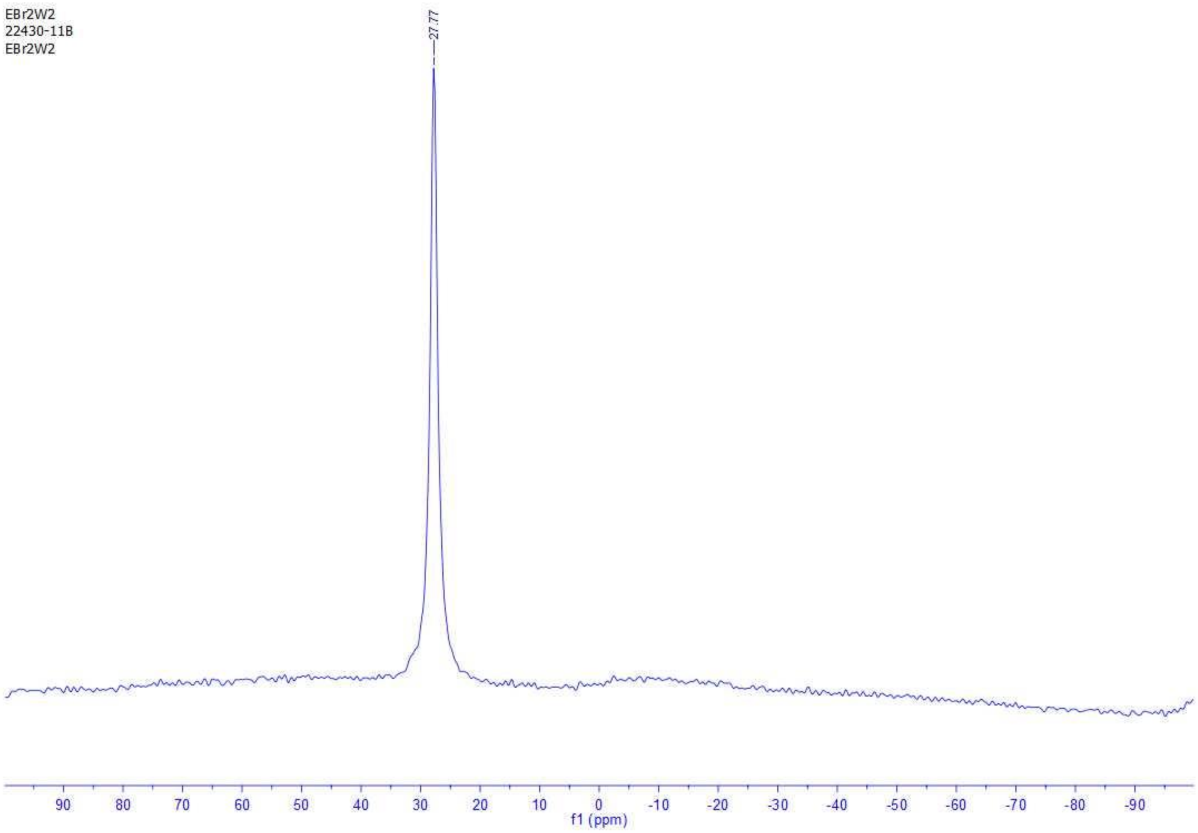

Fig. S5. [11]*B NMR spectrum of* ***1*** *in CDCl*$_3$

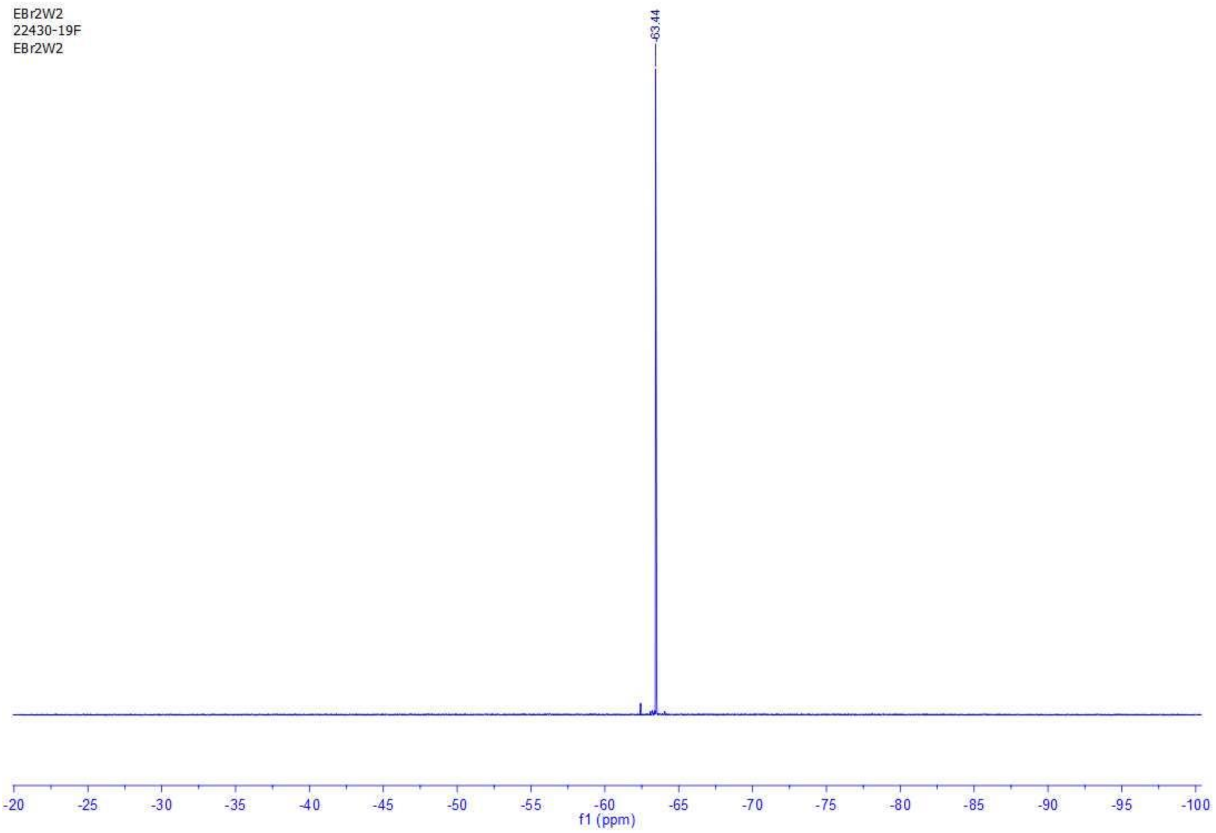

*Fig. S6. ^{19}F NMR spectrum of **1** in $CDCl_3$*

2. NMR spectra of **1** in C_6D_6

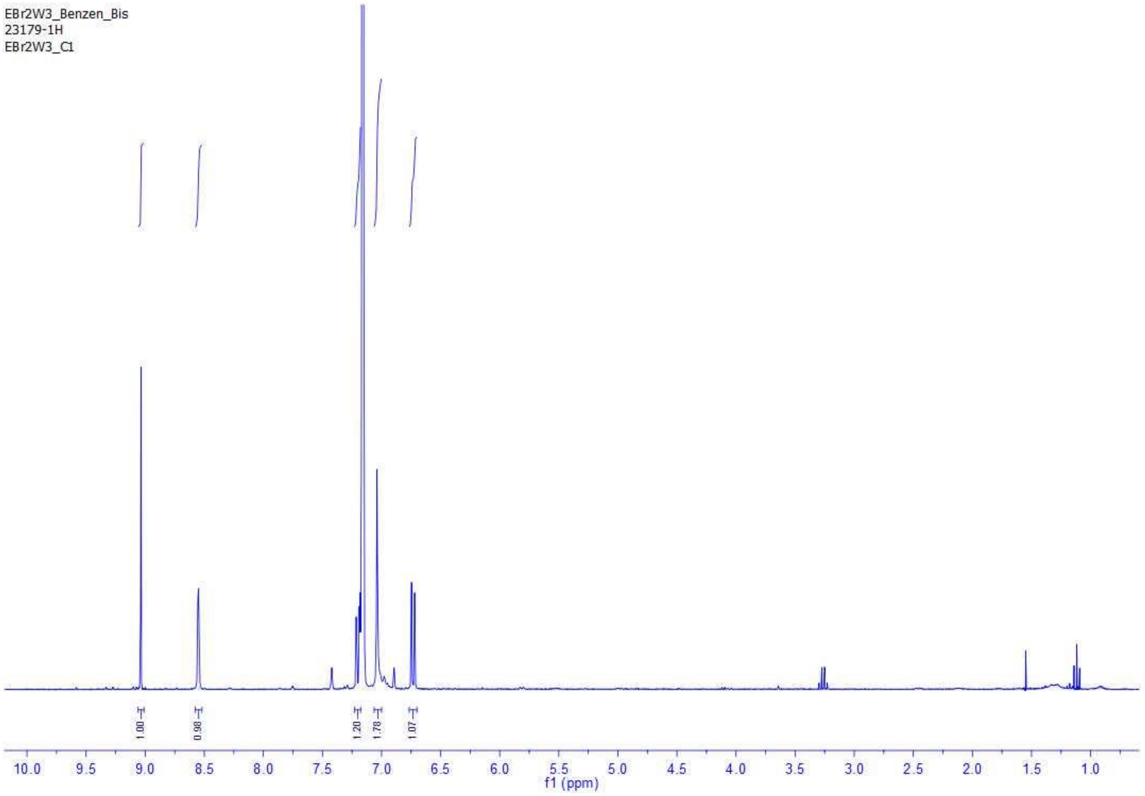

Fig. S7. [1]*H NMR spectrum of* ***1*** *in* C_6D_6

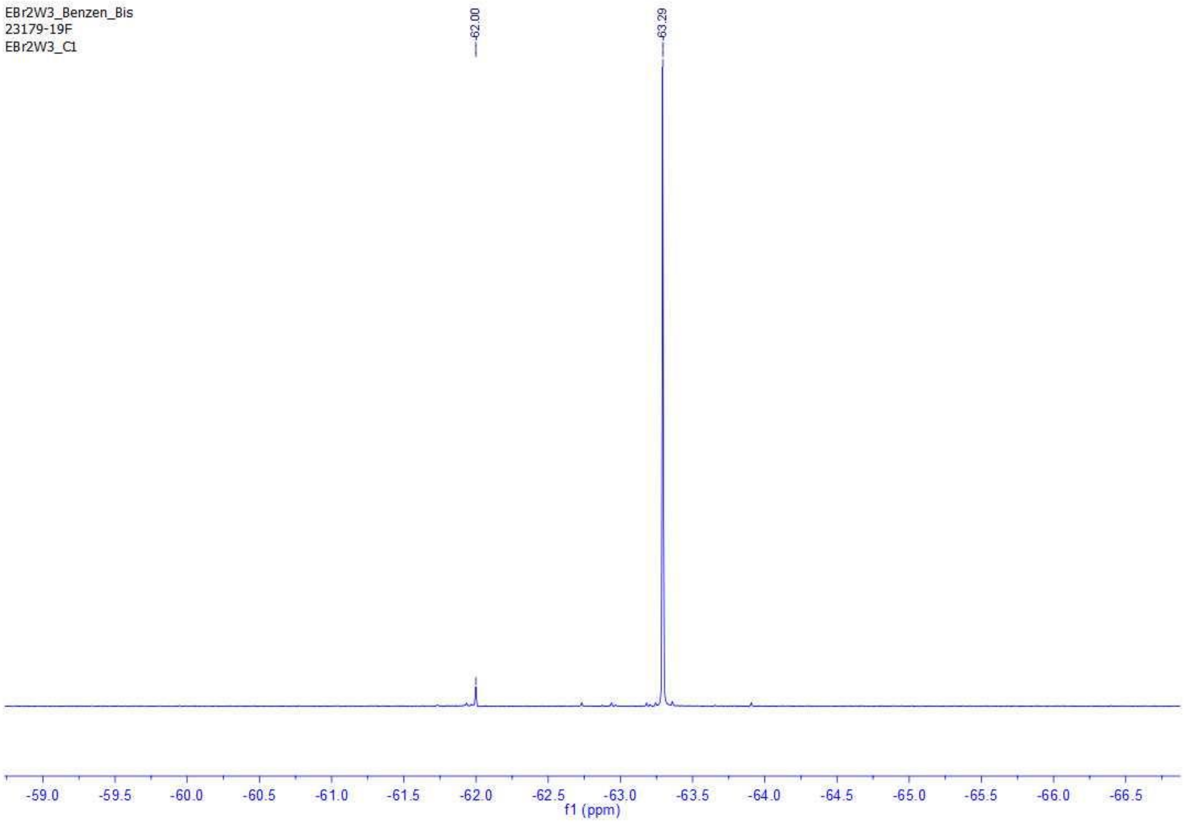

Fig. S8. [19]*F NMR spectrum of* ***1*** *in* C_6D_6

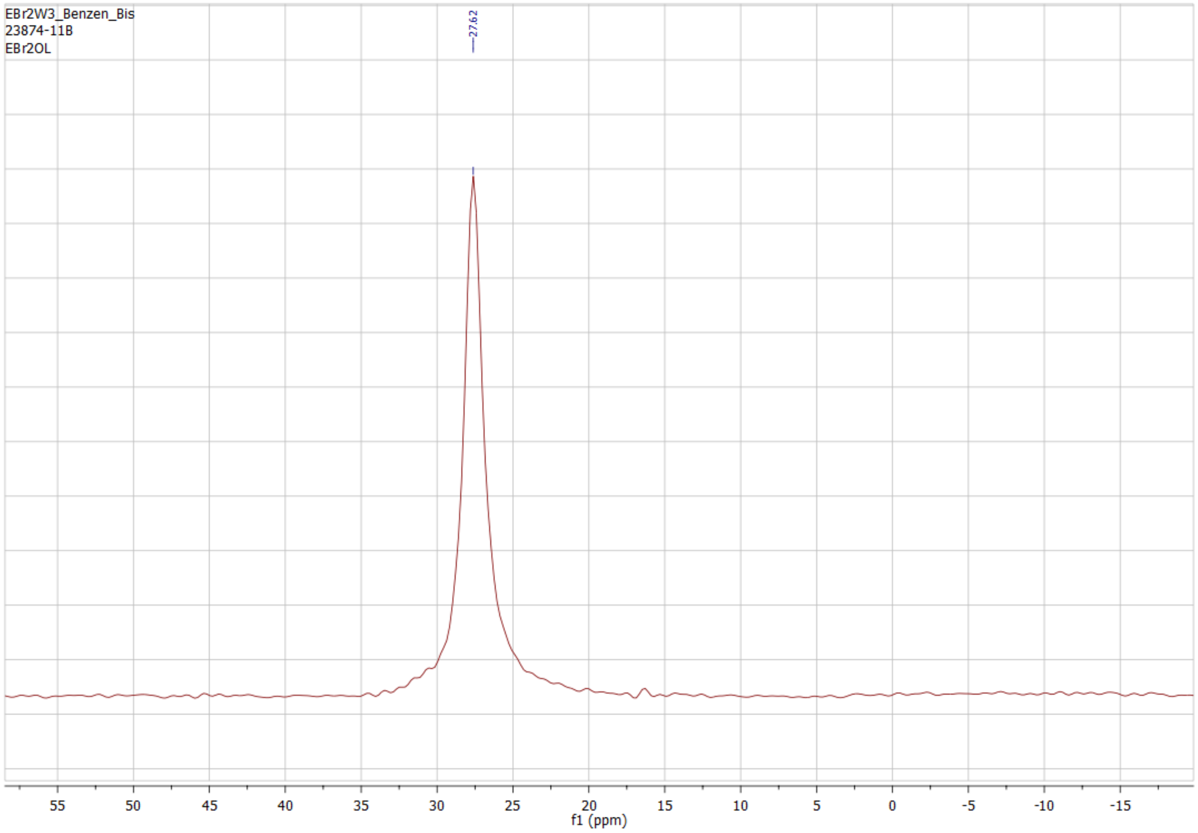

Fig. S9. *^{11}B NMR spectrum of* ***1*** *in C_6D_6*

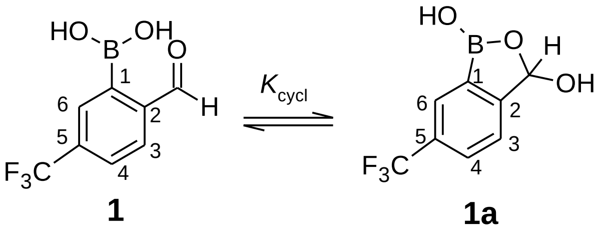

Scheme S1. The **1-1a** equilibrium with tom numbering.

3. NMR spectra of **1/1a** in DMSO-d_6

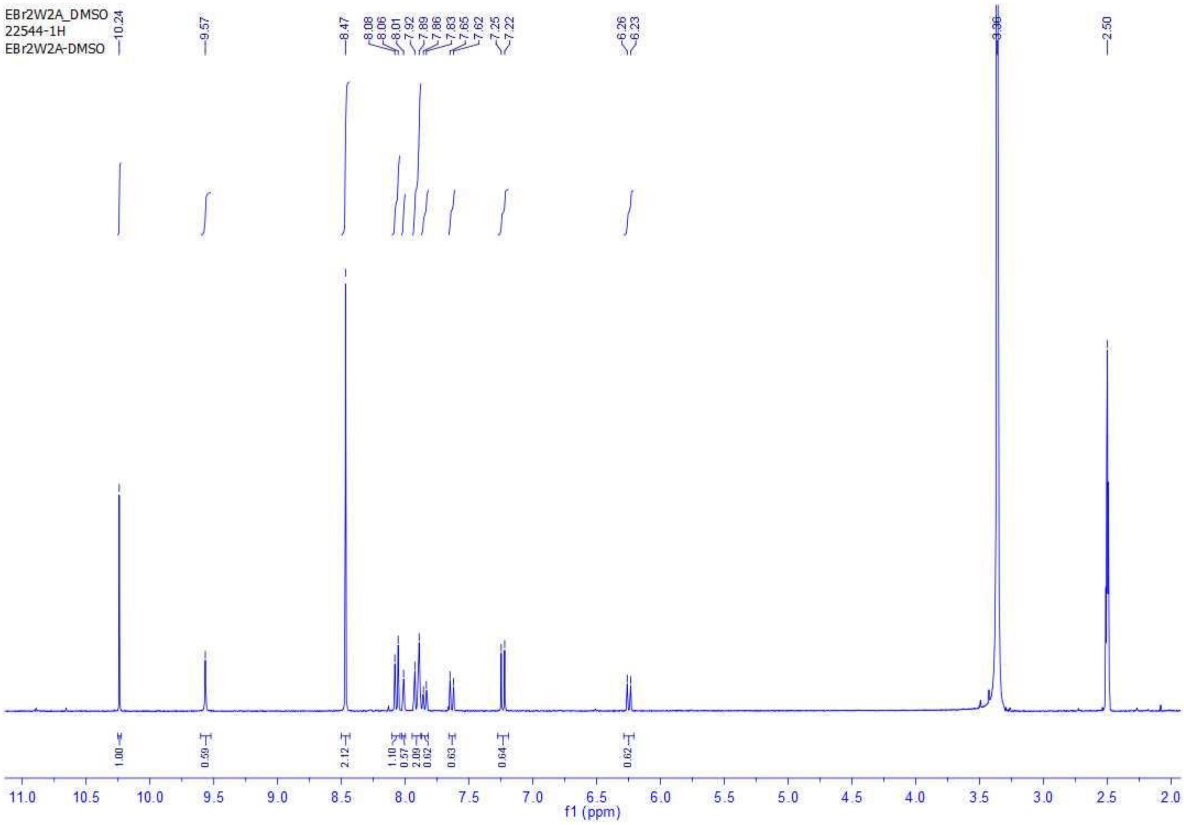

Fig. S10. ^{1}H *NMR spectrum of* ***1/1a*** *in DMSO-D*$_6$*, 2.3 mg dissolved in 0.5 ml of the solvent*

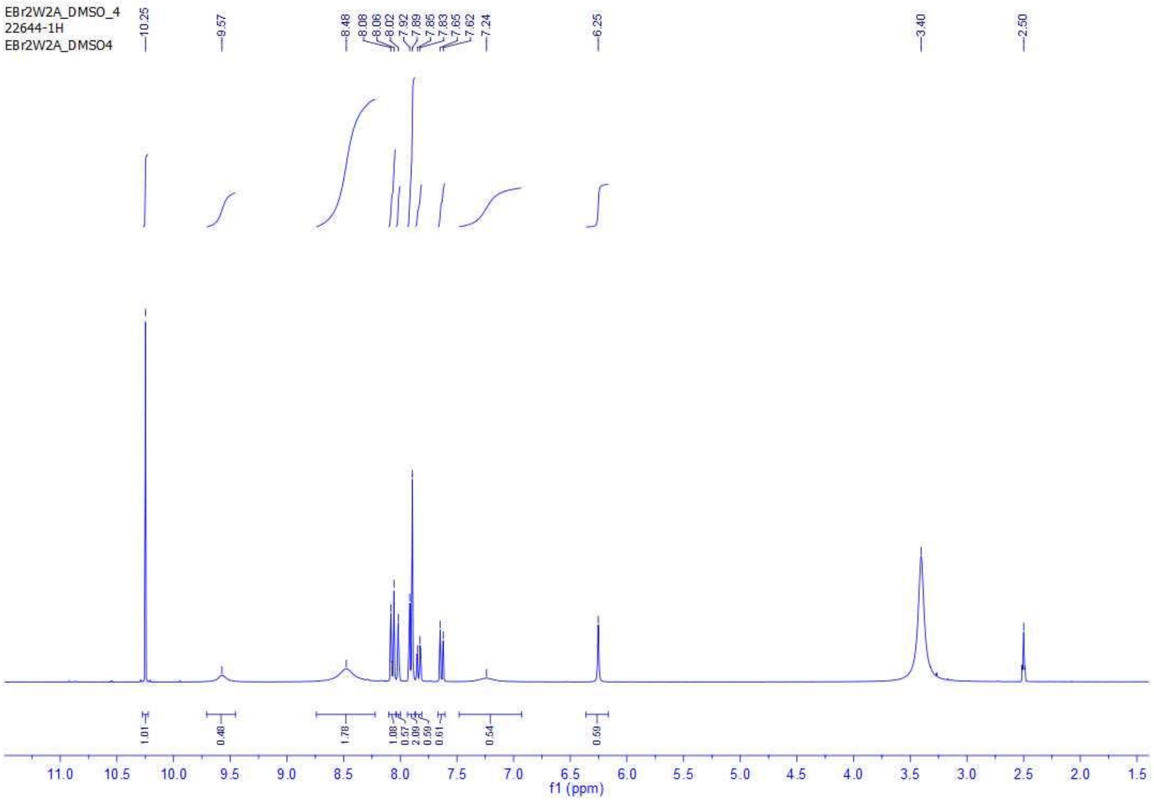

Fig. S11. ^{1}H *NMR spectrum of* ***1/1a*** *in DMSO-D*$_6$*, 29.7 mg dissolved in 0.5 ml of the solvent*

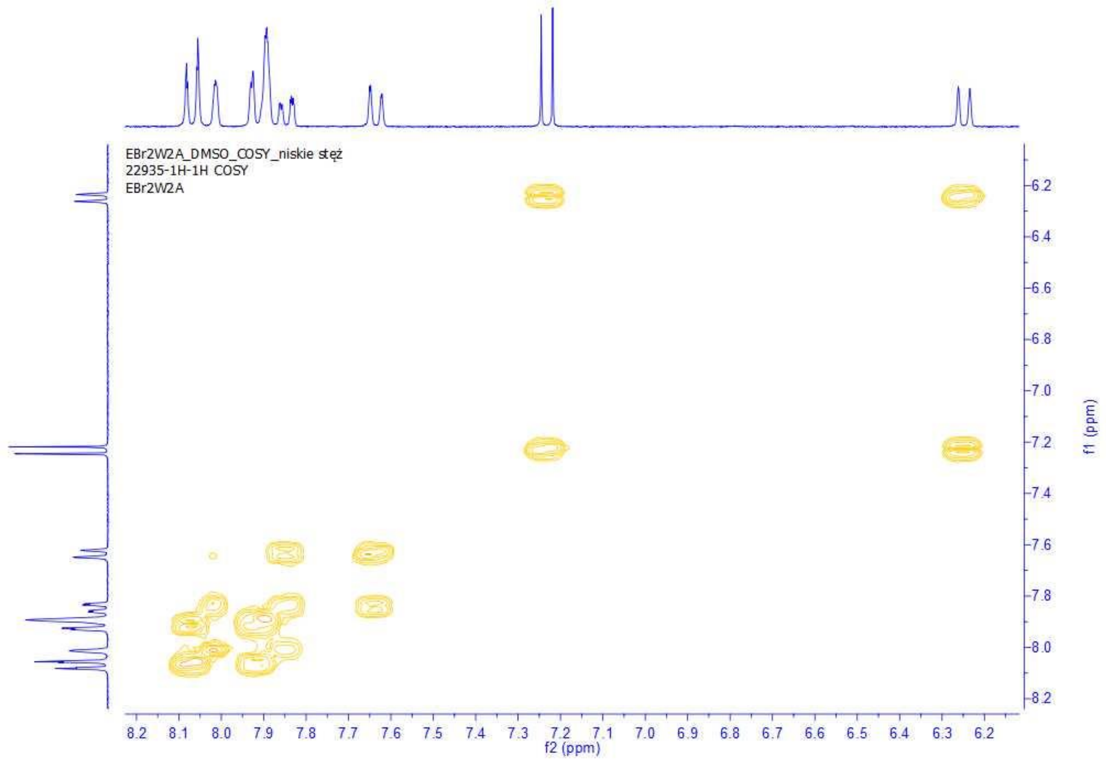

Fig. S12. [1]*H-*[1]*H COSY NMR spectrum of* ***1/1a*** *in DMSO-D*$_6$*, 2.3 mg dissolved in 0.5 ml of the solvent*

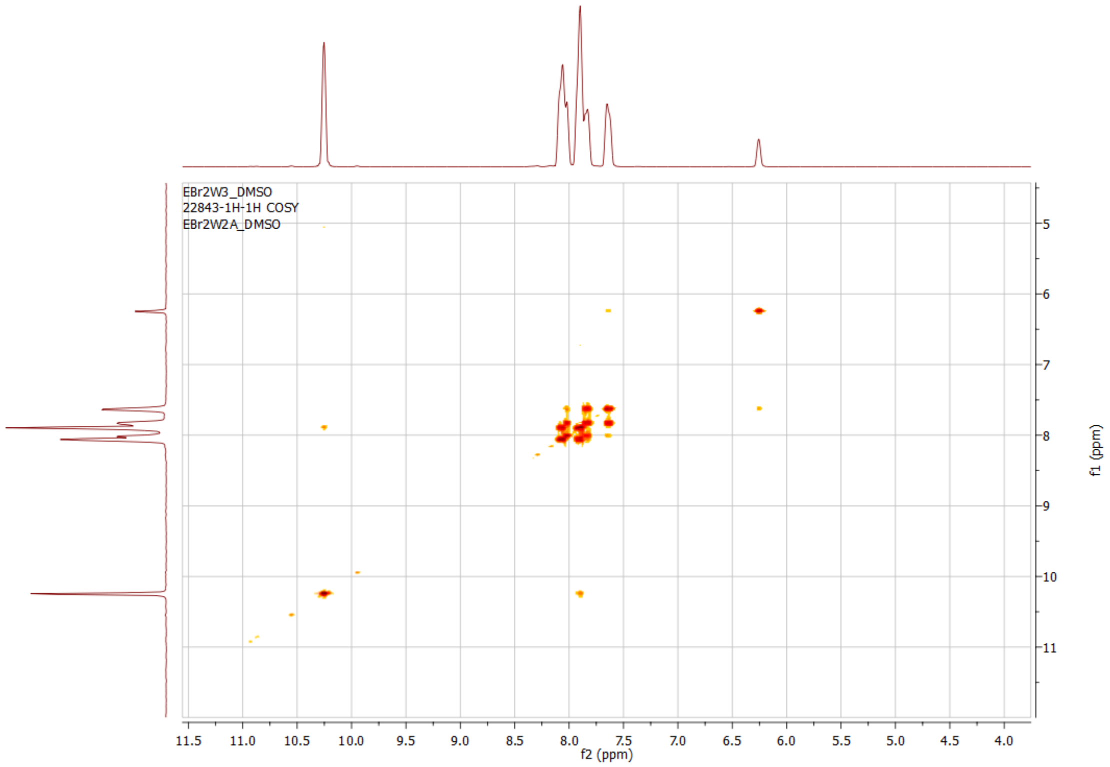

Fig. S13. [1]*H-*[1]*H COSY NMR spectrum of* ***1/1a*** *in DMSO-D*$_6$*, 29.7 mg dissolved in 0.5 ml of the solvent*

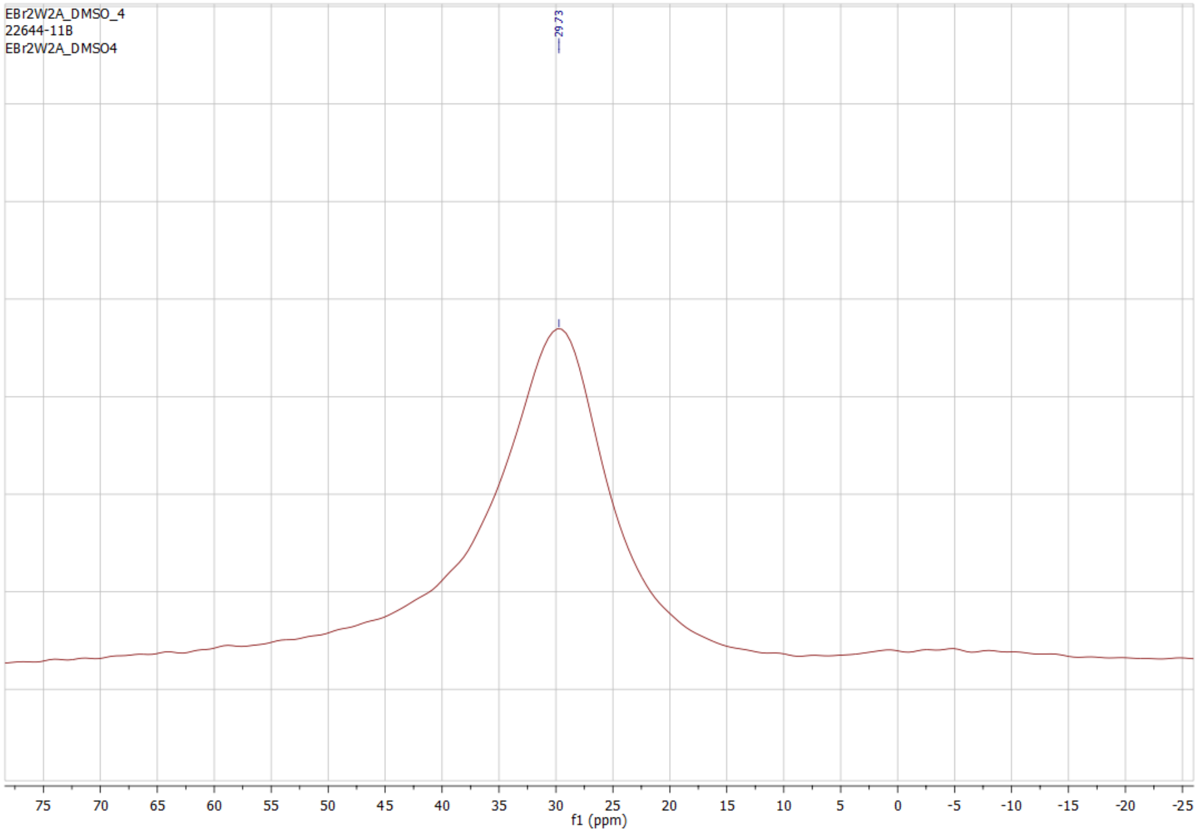

*Fig. S14. ^{11}B NMR spectrum of **1/1a** in DMSO-D$_6$*

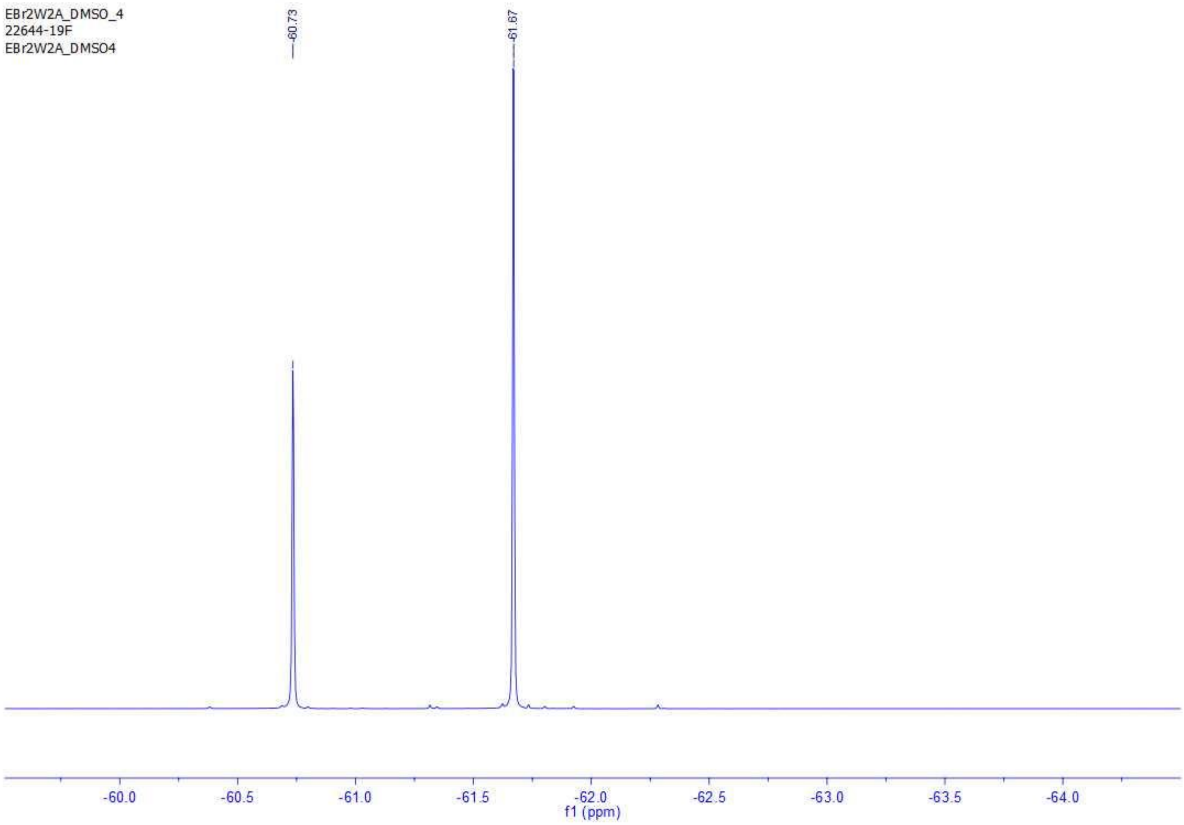

*Fig. S15. ^{19}F NMR spectrum of **1/1a** in DMSO-D$_6$*

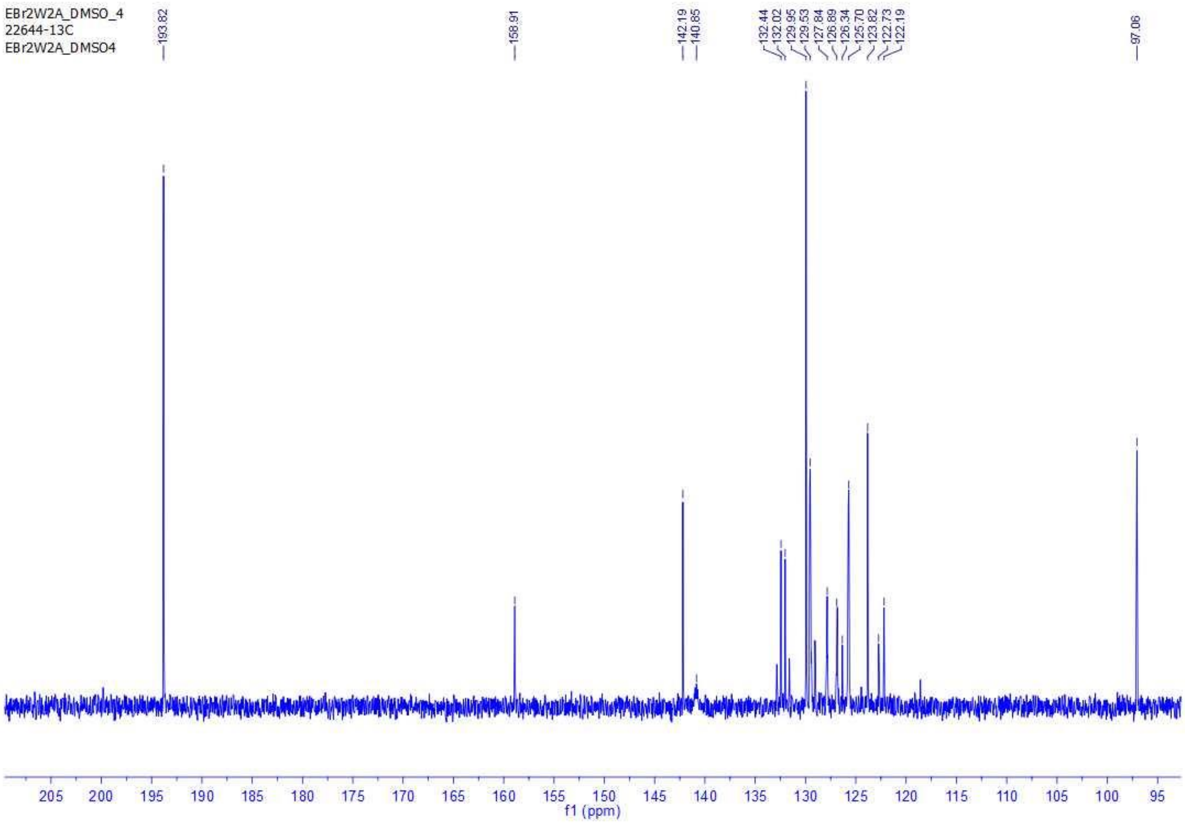

Fig. S16. *¹³C NMR spectrum of* ***1*/*1a*** *in DMSO-D₆*

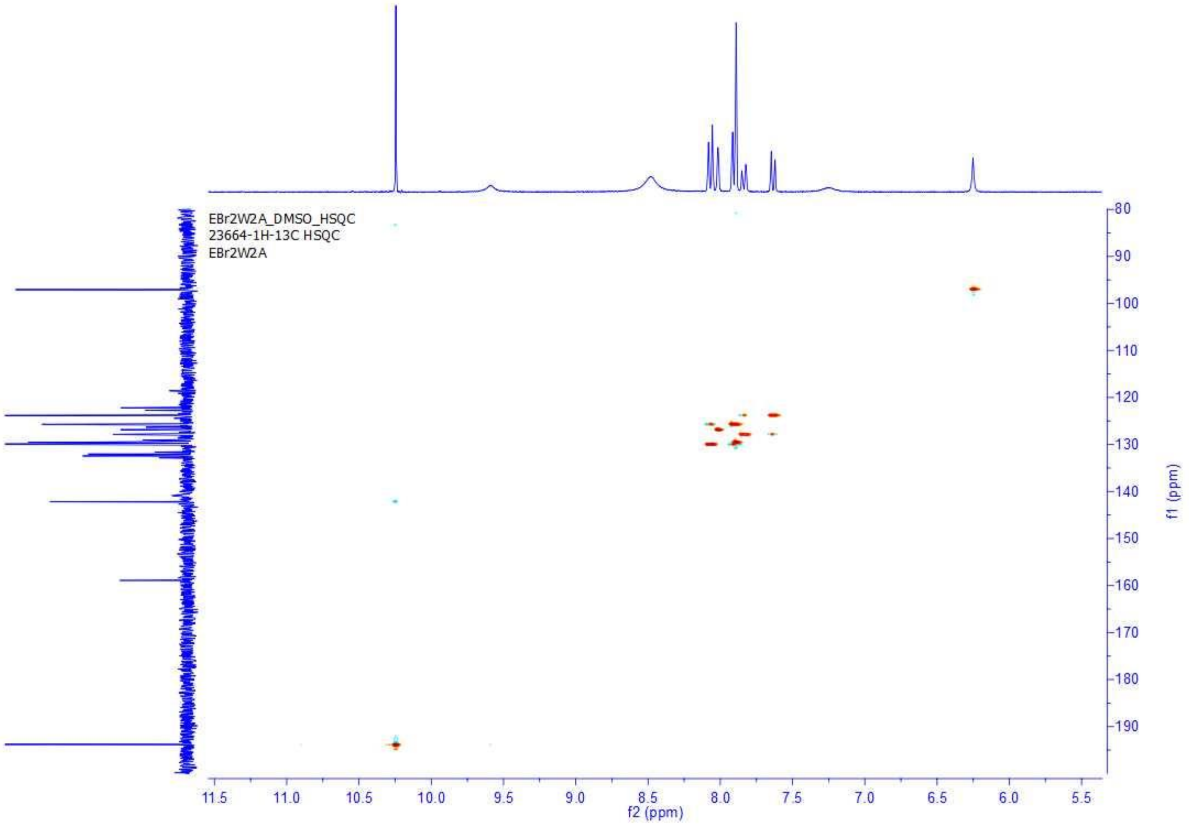

Fig. S17. *¹H - ¹³C HSQC NMR spectrum of* ***1*/*1a*** *in DMSO-D₆*

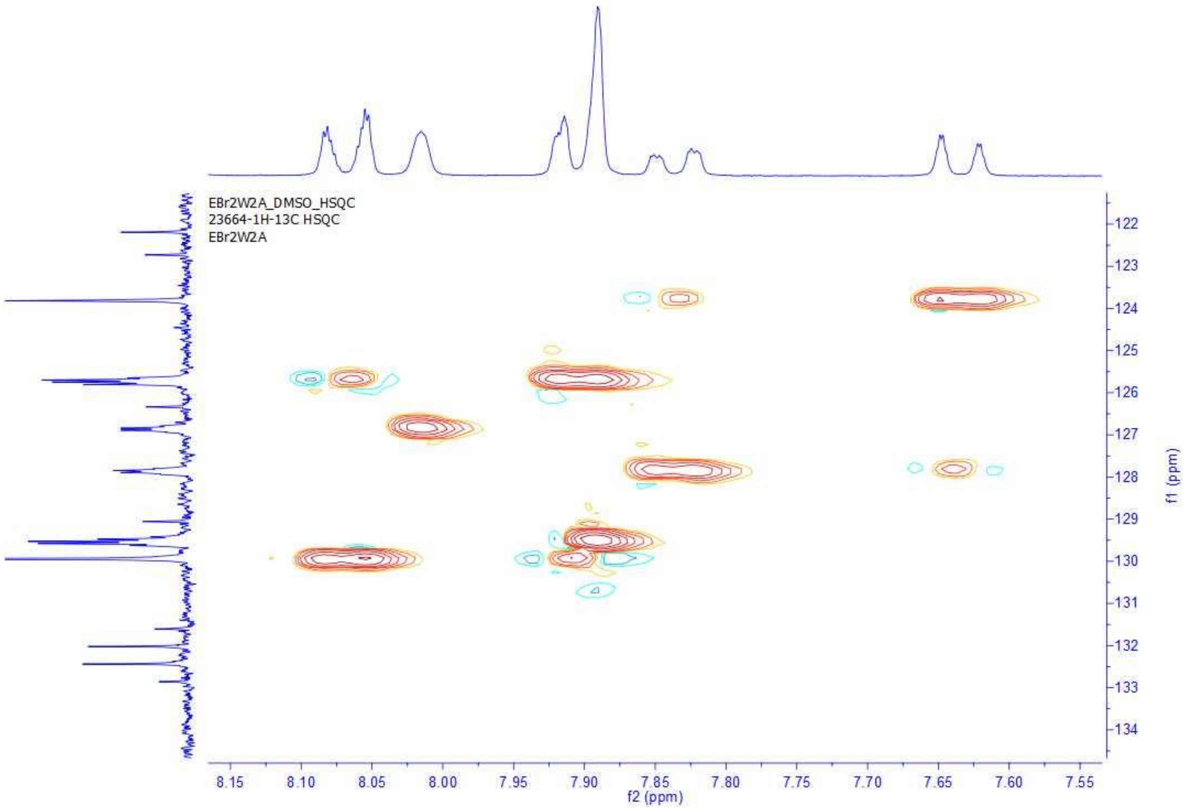

*Fig. S18. ^{1}H - ^{13}C HSQC NMR spectrum of **1/1a** in DMSO-D*$_6$ *(expanded)*

4. NMR spectra of **1/1a** in acetone-d$_6$

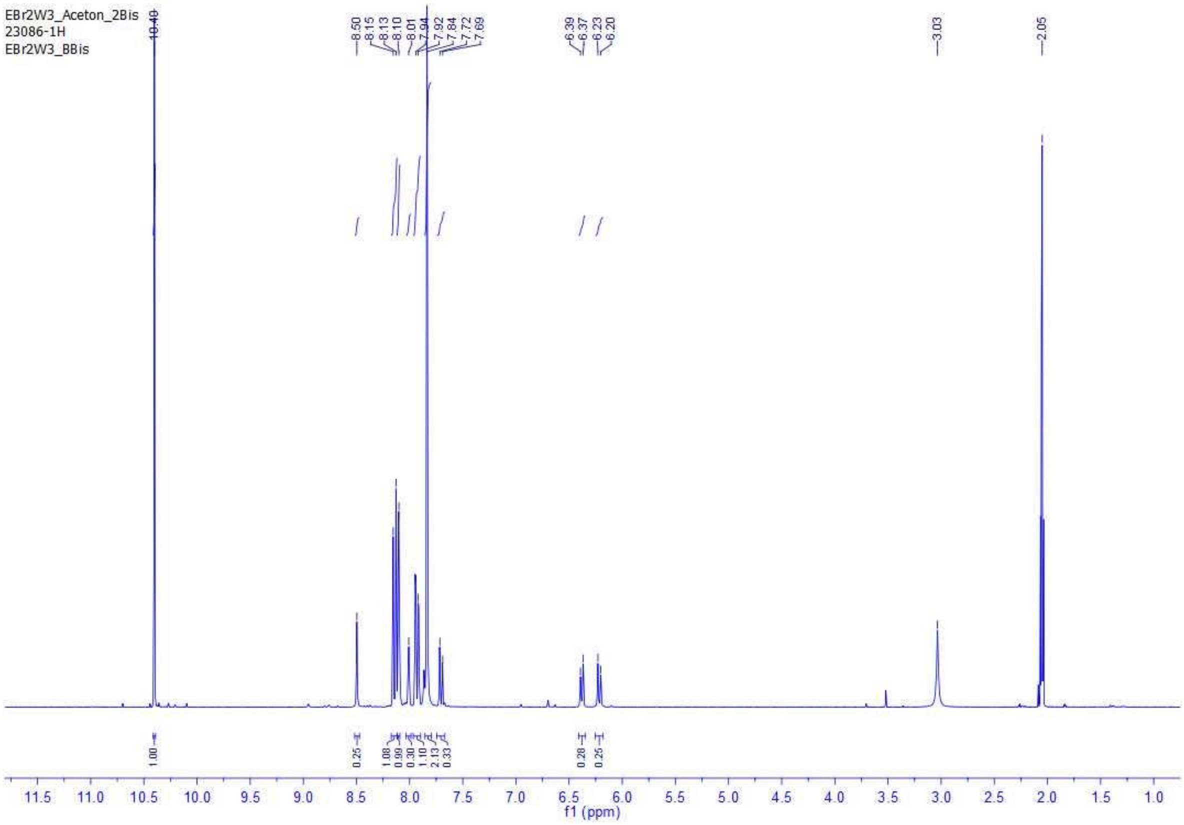

Fig. S19. ^{1}*H NMR spectrum of* ***1/1a*** *in acetone-D*$_6$*, 29.3 mg dissolved in 0.5 ml of the solvent*

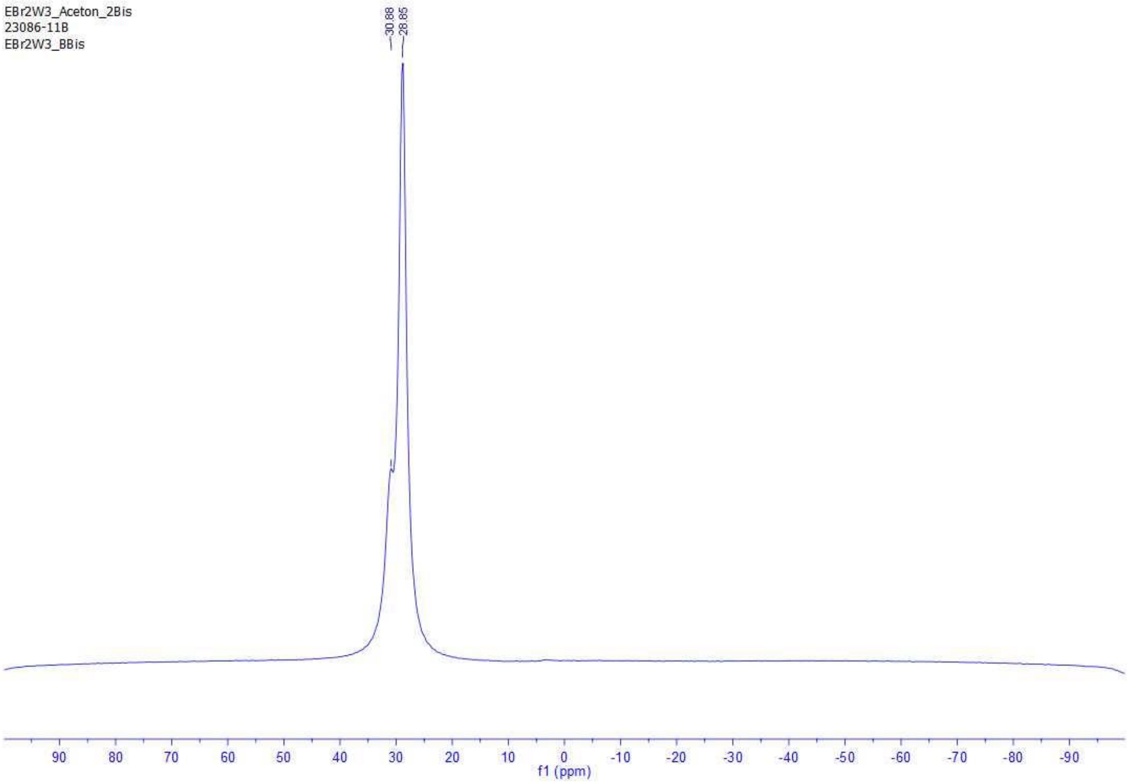

Fig. S20. ^{11}*B NMR spectrum of* ***1/1a*** *in acetone-D*$_6$*, 29.3 mg dissolved in 0.5 ml of the solvent*

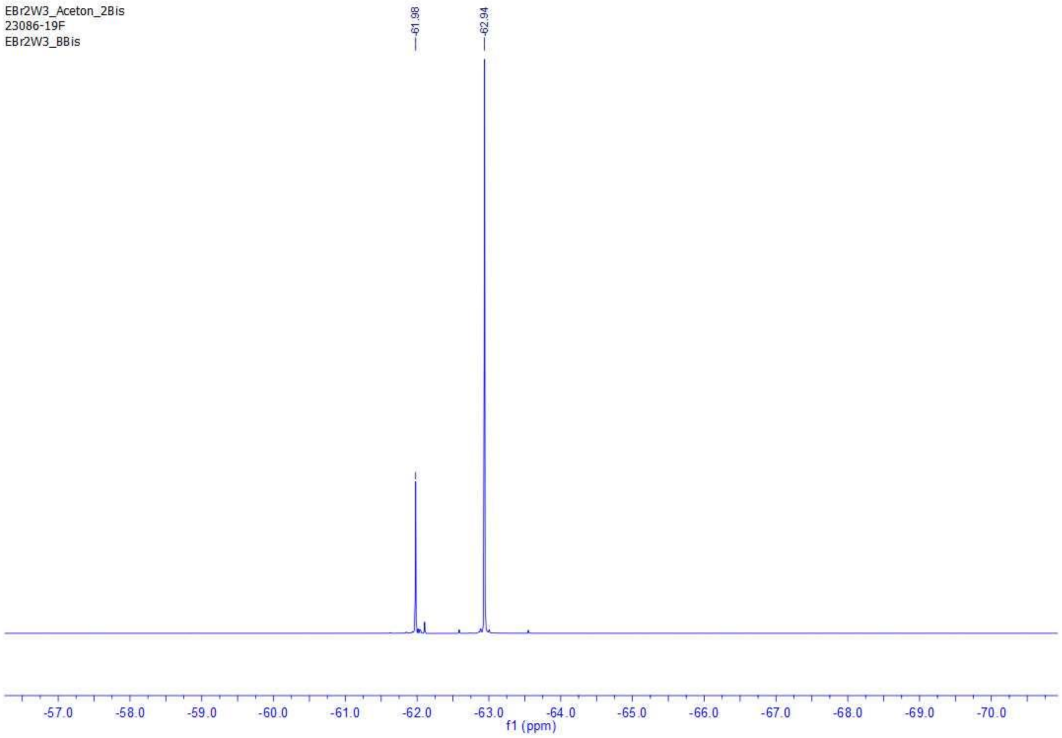

Fig. S21. *^{19}F NMR spectrum of* ***1/1a*** *in acetone-D6, 29.3 mg dissolved in 0.5 ml of the solvent*

5. NMR spectra of **1** in D_2O

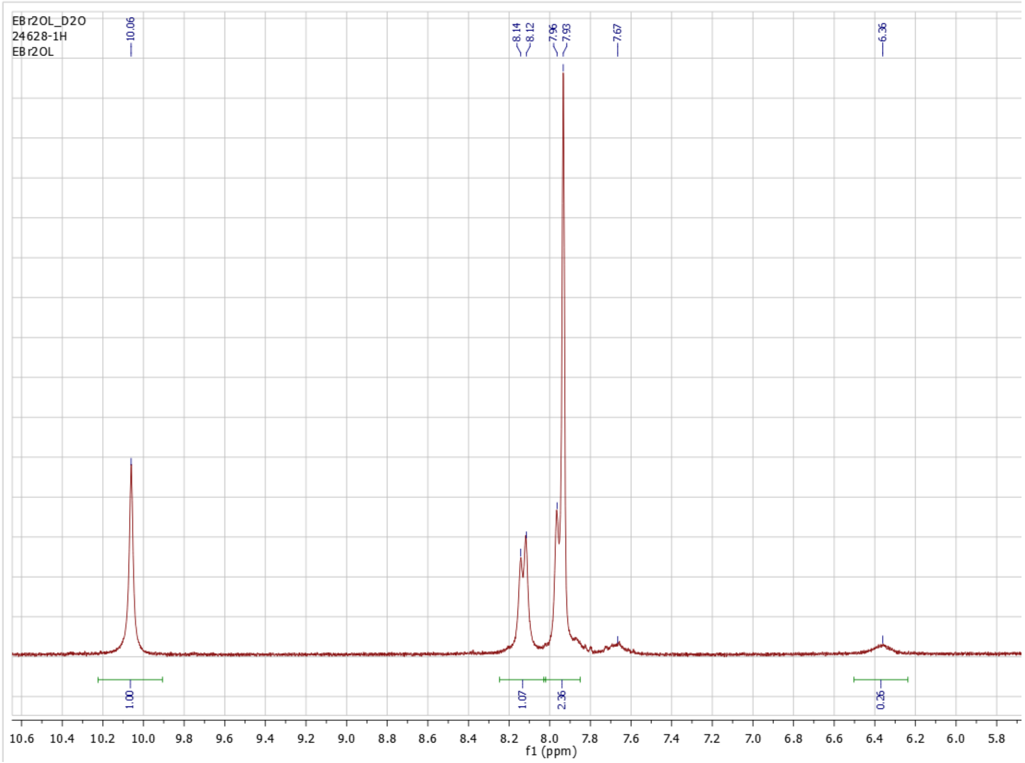

Fig. S22. *^{1}H NMR spectrum of* ***1/1a*** *in D_2O – saturated solution.*

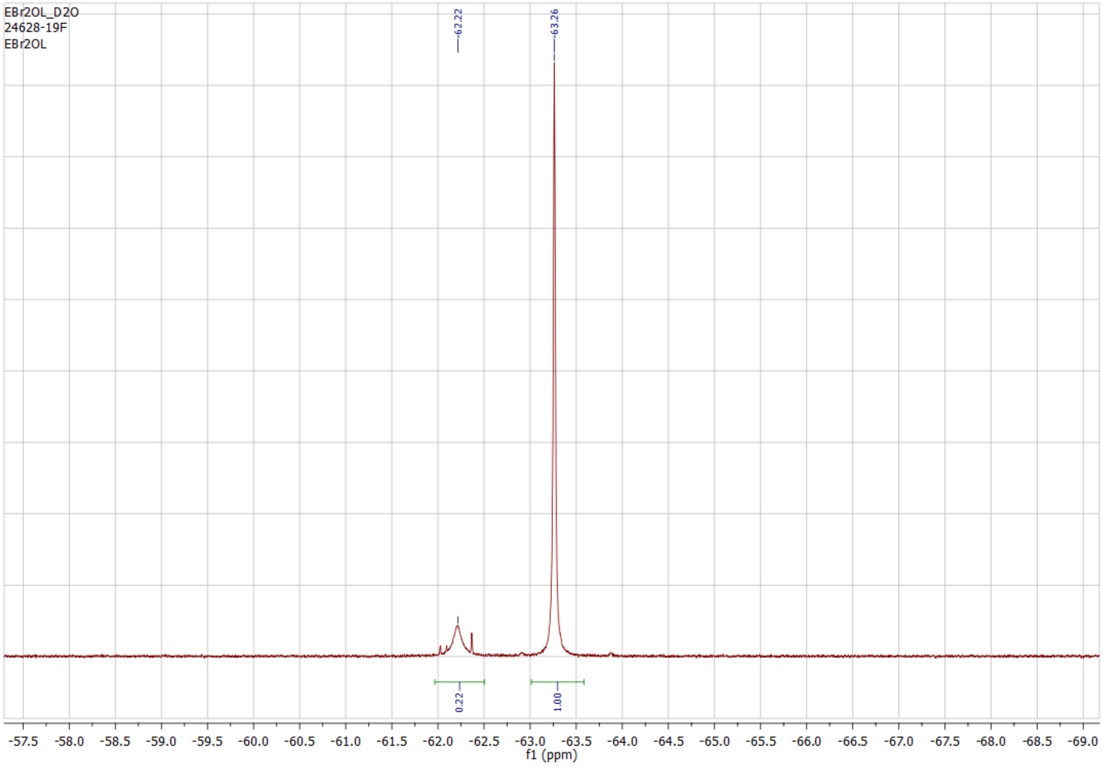

*Fig. S23. ^{19}F NMR spectrum of **1/1a** in D_2O – saturated solution.*

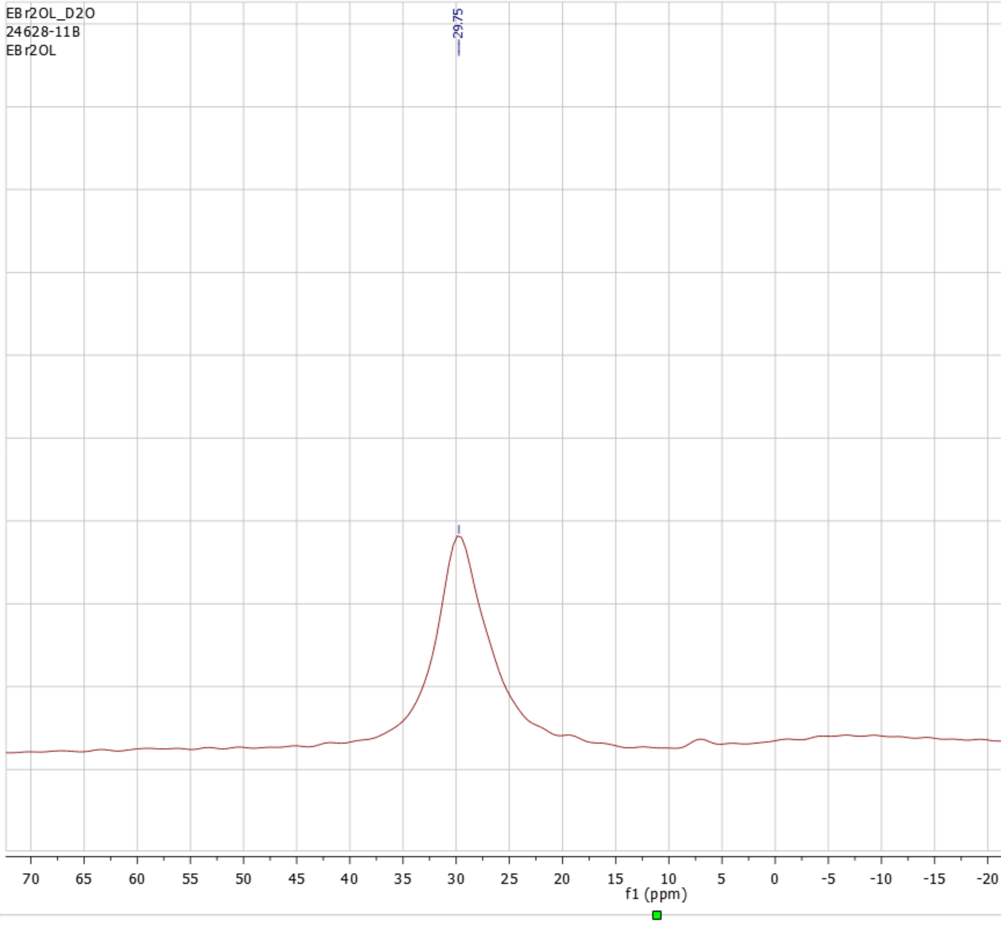

*Fig. S24. ^{11}B NMR spectrum of **1/1a** in D_2O – saturated solution.*

6. Selected bonds lengths and torsional angles in **1**.

Table S1. Bond lengths in molecules **1_I** and **1_II** (Å).

Bond*	**Molecule 1_I**	**Molecule 1_II**
O1–B1	1.3463(17)	1.3429(17)
O2–B1	1.3468(16)	1.3468(16)
O3–C7	1.1954(17)	1.2013(17)
C1–B1	1.5935(18)	1.5940(17)
C1–C2	1.3909(17)	1.3905(16)
C1–C6	1.4141(16)	1.4156(16)
C2–C3	1.3864(17)	1.3856(16)
C3–C4	1.377(2)	1.3764(19)
C4–C5	1.377(2)	1.375(2)
C5–C6	1.3942(18)	1.3918(18)
C6–C7	1.4730(18)	1.4728(18)
C3–C8	1.4938(19)	1.4970(18)

*Labels for **1_II** are derived from these for **1_I** by adding _1.

Table S2. Selected bond and torsional angles for molecules **1_I** and **1_II** (°).

Angle*	**Molecule 1_I**	**Molecule 1_II**
O1–B1–O2	118.64(11)	119.14(11)
O1–B1–C1–	115.32(11)	115.06(11)
O2–B1–C1	126.03(11)	125.79(11)
C2–C1–B1	114.58(10)	114.96(10)
C6–C1–B1	129.13(11)	128.72(11)
C2–C1–C6	116.28(11)	116.32(10)
C1–C6–C7	125.80(11)	126.00(11)
C5–C6–C7	113.74(11)	113.69(11)
O3–C7–C6	128.18(12)	127.32(13)
C2–C1–B1–O1	2.16(18)	6.38(18)
C2–C1–B1–O2	-176.77(14)	-172.69(14)
C6–C1–B1–O1	-179.29(13)	-173.54(13)
C6–C1–B1–O2	1.8(2)	7.4(2)
C1–C6–C7–O3	4.8(3)	-7.2(3)
B1–C1–C6–C7	0.1(2)	5.8(2)
C2–C1–C6–C7	178.67(13)	174.31(13)
C4–C5–C6–C7	-179.26(13)	-174.53(13)
C1–C2–C3–C8	-178.60(12)	-175.85(12)
C8–C3–C4–C5	178.04(13)	175.91(13)

*Labels for **1_II** are derived from these for **1_I** by adding _1.

7. Acidity constant determination

First, the absorption maxima wavelengths of the both form of the boronic acid were chosen (247 nm and 271 nm). The values of absorbance from each scan for this wavelengths were collected to form sigmoidal curve. The sigmoidal curve was also derivatised.

For acidity constant value determination, data modelling by OriginPro 8 software was proceeded.

One method consist in first derivative curve modelling by Gauss function (Eq. 1).

$$y = y_0 + \frac{A}{w\sqrt{\frac{\pi}{2}}} e^{-2\frac{(x-x_c)^2}{w^2}}$$

Where: A – Area, w – width, x_c – center, y_0 – offset

Eq. 1. The formula of the Gauss function

The second method case is modelling of sigmoidal curve by two functions: Boltzmann function (Eq. 2) and Biphasic Dose Response (BDR) function (Eq. 3). Although there is one equilibrium, the second function (for two equilibria created) found to be the most fitting to experimental points.

$$y = \frac{A_1 - A_2}{1 + e^{\frac{(x-x_0)}{dx}}} + A_2$$

Where: A_1 – initial value, A_2 – final value, x_c – center

Eq. 2. The formula of the Boltzmann function

$$y = A_1 + (A_2 - A_1) \times$$

Where: A_1 – initial value, A_2 – final value, p – proportion, h – curve slope, log x – first and second inflection point

Eq. 3. The formula of the BDR function

Table S3. The values of the acidity constant determined by Gauss, Boltzmann and BDR function

**Seria**	**Gauss (R^2)**	**Boltzmann (R^2)**	**BDR (R^2)**
A	5.67 (0.97077)	5.65 (0.99919)	5.66 (0.99993)
B	5.65 (0.96559)	5.67 (0.99937)	5.68 (0.99997)
C	5.69 (0.97531)	5.64 (0.99879)	5.66 (0.99996)
**pKa**	**5.67 (±0.02)**	**5.65 (±0.02)**	**5.67 (±0.01)**

REFERENCES:

1. Kowalska, K.; Adamczyk-Woźniak, A.; Gajowiec, P.; Gierczyk, B.; Kaczorowska, E.; Popenda, Ł.; Schroeder, G.; Sikorski, A.; Sporzyński, A. Fluoro-substituted 2-formylphenylboronic acids: Structures, properties and tautomeric equilibria. *J. Fluor. Chem.***2016**, *187*, 1–8, doi: 10.1016/j.jfluchem.2016.05.001.

8. Docking studies – input and optimal structures

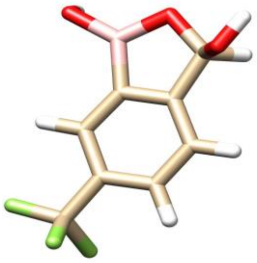

*Fig. S25. Structure **1a-S***

*Table S4. Coordinates of structure **1a-S***

	Coordinates of the input structure **(1a-S)** – prior to optimization			Coordinates of the optimized structure **(1a-S)**		
C	-4.72200	-0.64700	-0.01400	0.91100	0.55600	-0.08500
C	-4.43000	-3.39400	-0.00200	-0.97900	-1.48100	-0.16500
C	-3.29500	-2.56700	-0.06100	0.36600	-1.80800	-0.25500
C	-3.47500	-1.18300	-0.06500	1.29400	-0.77700	-0.22000
C	-4.49400	0.85000	-0.04100	-0.44100	0.87900	0.00400
C	-5.87400	-1.42800	0.04600	-1.37900	-0.14600	-0.04000
C	-5.72900	-2.83400	0.05300	2.79800	-0.88900	-0.30200
C	-3.05700	1.15900	-0.10900	-2.85200	0.14400	0.05500
B	-5.54300	1.86400	-0.00000	2.25000	1.35100	-0.04900
O	-2.41600	-0.11700	-0.12400	3.29400	0.45800	-0.17400
O	-6.84600	-0.95400	0.08500	2.42900	2.68500	0.07100
O	-6.92500	-3.74900	0.11600	3.27100	-1.70500	0.72300
F	-4.29900	-4.46900	0.00200	-3.11600	1.45900	0.15700
F	-2.30200	-2.99600	-0.10300	-3.40900	-0.45800	1.12900
F	-8.11200	-3.03900	0.16300	-3.52100	-0.31400	-1.02700
H	-6.83900	-4.54100	1.24700	-0.76400	1.90600	0.11100
H	-6.94200	-4.56200	-1.00300	-1.73100	-2.26000	-0.18800
H	-1.57300	-0.24000	0.98800	0.67700	-2.84300	-0.33800
H	-0.83000	0.40200	0.84400	4.22300	-1.81000	0.60900
H	-1.84200	-0.24700	-1.07000	3.13800	-1.24800	-1.28000
H	-5.76800	2.02900	-0.95100	3.35900	2.93800	0.07600

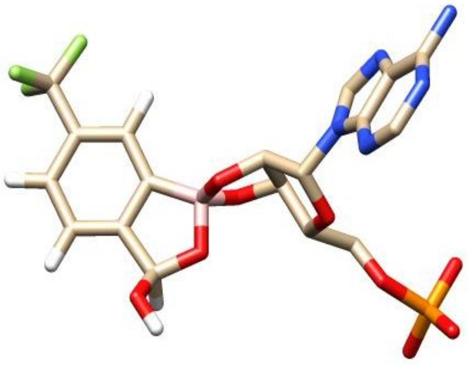

*Fig. S26 The **1a-S-AMP** spiroester structure (structure A)*

Table S5: Coordinates of 1a-S-AMP spiroester

C	26.027	59.041	10.864
C	26.530	58.442	8.196
C	25.724	59.529	8.501
C	25.479	59.808	9.838
B	25.535	59.724	12.175
C	26.835	57.950	10.553
C	27.078	57.656	9.216
O	24.747	60.805	11.837
C	24.640	60.929	10.405
H	27.273	57.340	11.331
C	27.945	56.492	8.822
H	26.743	58.195	7.164
H	25.310	60.146	7.712
F	28.402	55.803	9.883
F	29.023	56.892	8.113
F	27.277	55.617	8.036
O	25.125	62.157	9.963
H	24.535	62.848	10.288
H	23.576	60.817	10.168
P	21.391	61.754	17.311
O	21.558	60.568	18.234
O	20.203	61.660	16.393
O	21.645	63.099	17.972
N	27.150	60.576	16.551
C	27.152	59.299	16.988
N	27.829	59.205	18.153
C	28.259	60.452	18.461
C	29.027	61.043	19.563
N	29.478	60.251	20.569
N	29.264	62.370	19.534
C	28.812	63.143	18.528
N	28.094	62.668	17.497
C	27.799	61.347	17.421
O	22.714	61.636	16.383
C	23.085	60.383	15.781
C	24.250	60.671	14.847
O	25.183	61.468	15.587
C	24.992	59.441	14.343
O	24.662	59.085	13.007
C	26.407	59.944	14.280
O	26.522	60.388	12.944
C	26.511	61.070	15.307

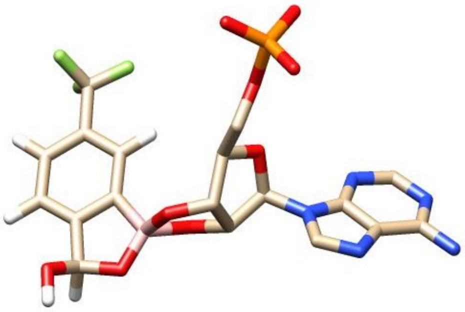

*Fig. S27. The **1a-S-AMP** spiroester structure (structure B)*

Table S6: Coordinates of 1a-S-AMP spiroester

C	25.075	60.771	10.936
C	24.308	62.312	8.753
C	24.779	61.025	8.536
C	25.164	60.274	9.638
B	25.561	59.600	11.842
C	24.602	62.063	11.149
C	24.224	62.828	10.051
O	25.900	58.533	11.035
C	25.703	58.863	9.646
H	24.523	62.472	12.148
C	23.704	64.228	10.222
H	23.999	62.926	7.917
H	24.832	60.620	7.532
F	23.683	64.621	11.508
F	22.446	64.353	9.747
F	24.456	65.125	9.544
O	24.790	58.001	9.044
H	25.185	57.122	9.007
H	26.688	58.788	9.172
P	21.391	61.754	17.311
O	21.558	60.568	18.234
O	20.203	61.660	16.393
O	21.645	63.099	17.972
N	27.150	60.576	16.551
C	27.152	59.299	16.988
N	27.829	59.205	18.153
C	28.259	60.452	18.461
C	29.027	61.043	19.563
N	29.478	60.251	20.569
N	29.264	62.370	19.534
C	28.812	63.143	18.528
N	28.094	62.668	17.497
C	27.799	61.347	17.421
O	22.714	61.636	16.383
C	23.085	60.383	15.781
C	24.250	60.671	14.847
O	25.183	61.468	15.587
C	24.992	59.441	14.343
O	24.662	59.085	13.007
C	26.407	59.944	14.280
O	26.522	60.388	12.944
C	26.511	61.070	15.307

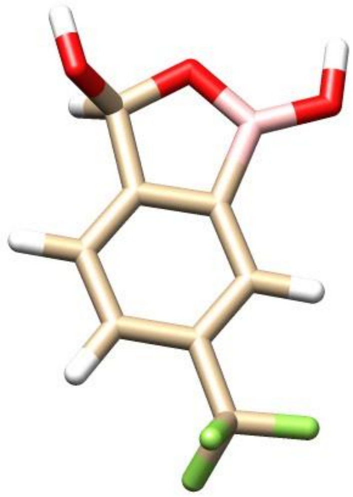

Fig. S28. Structure 1a-R

Table S7: Coordinates of structure 1a-R

	Coordinates of the input structure (**1a-R**) – prior to optimization			Coordinates of the optimized structure (**1a-R**)		
C	-4.71300	-0.63700	-0.00700	-0.911	0.556	-0.086
C	-4.41000	-3.38300	-0.01200	0.979	-1.481	-0.166
C	-3.27900	-2.55100	-0.07100	-0.366	-1.809	-0.255
C	-3.46400	-1.16700	-0.06900	-1.294	-0.777	-0.220
C	-4.49100	0.86100	-0.02200	0.441	0.879	0.003
C	-5.86200	-1.42300	0.05300	1.379	-0.145	-0.041
C	-5.71100	-2.82800	0.05100	-2.798	-0.889	-0.301
C	-2.40900	-0.09300	-0.12500	2.852	0.144	0.055
B	-4.49100	0.86100	-0.02200	-2.250	1.351	-0.050
O	-3.05500	1.17800	-0.08900	-3.294	0.458	-0.174
O	-5.54500	1.87000	0.03600	-2.428	2.685	0.071
O	-1.67400	-0.20100	-1.31400	-3.271	-1.705	0.723
F	-8.09200	-3.04300	0.17000	3.116	1.460	0.146
F	-6.80900	-4.54600	1.24000	3.523	-0.324	-1.020
F	-6.92200	-4.55500	-1.01000	3.405	-0.449	1.136
H	-6.83500	-0.95300	0.10100	-3.138	-1.247	-1.280
H	-4.27500	-4.45800	-0.01400	-3.358	2.939	0.077
H	-2.28500	-2.97700	-0.11900	-4.223	-1.811	0.608
H	-1.74100	-0.17600	0.76400	0.764	1.906	0.109
H	-5.77400	2.04600	-0.91200	1.731	-2.260	-0.189
H	-0.99400	-0.90900	-1.16700	-0.677	-2.843	0.337

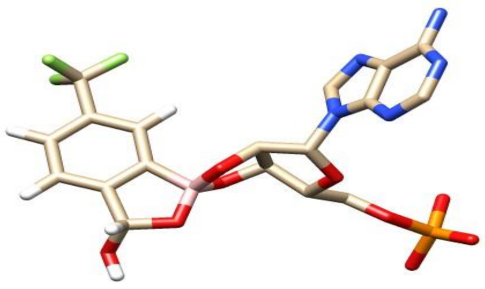

*Fig. S29 The **1a-R-AMP** spiroester structure (structure A)*

*Table S8: Coordinates of **1a-R-AMP** spiroester (structure A)*

C	26.223	58.520	11.445
C	27.140	56.633	9.623
C	26.512	57.775	9.148
C	26.067	58.709	10.073
B	25.554	59.768	12.095
C	26.854	57.371	11.916
C	27.313	56.435	10.998
O	25.077	60.584	11.089
C	25.363	60.016	9.796
H	26.986	57.201	12.976
C	27.998	55.174	11.449
H	27.500	55.882	8.930
H	26.366	57.922	8.085
F	28.129	55.106	12.786
F	29.236	55.063	10.921
F	27.319	54.073	11.058
H	26.010	60.738	9.283
O	24.197	59.785	9.072
H	23.804	60.638	8.853
P	21.391	61.754	17.311
O	21.558	60.568	18.234
O	20.203	61.660	16.393
O	21.645	63.099	17.972
N	27.150	60.576	16.551
C	27.152	59.299	16.988
N	27.829	59.205	18.153
C	28.259	60.452	18.461
C	29.027	61.043	19.563
N	29.478	60.251	20.569
N	29.264	62.370	19.534
C	28.812	63.143	18.528
N	28.094	62.668	17.497
C	27.799	61.347	17.421
O	22.714	61.636	16.383
C	23.085	60.383	15.781
C	24.250	60.671	14.847
O	25.183	61.468	15.587
C	24.992	59.441	14.343
O	24.662	59.085	13.007
C	26.407	59.944	14.280
O	26.522	60.388	12.944
C	26.511	61.070	15.307

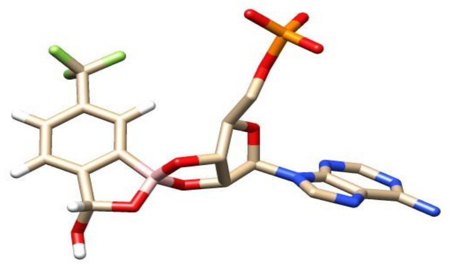

*Fig. S30. The **1a-R-AMP** spiroester structure (structure B)*

*Table S9. Coordinates of the **1a-R-AMP** spiroester structure (structure B)*

C	24.892	60.836	11.232
C	24.147	62.467	9.108
C	24.863	61.308	8.846
C	25.220	60.504	9.920
C	24.173	62.001	11.489
C	23.802	62.809	10.421
O	26.135	58.799	11.256
C	25.993	59.206	9.881
H	23.907	62.282	12.499
C	23.028	64.080	10.638
H	23.852	63.119	8.295
H	25.144	61.050	7.832
F	22.732	64.291	11.934
F	21.861	64.076	9.958
F	23.716	65.160	10.206
H	25.436	58.404	9.382
O	27.231	59.407	9.278
H	27.674	58.553	9.208
P	21.391	61.754	17.311
O	21.558	60.568	18.234
O	20.203	61.660	16.393
O	21.645	63.099	17.972
N	27.150	60.576	16.551
C	27.152	59.299	16.988
N	27.829	59.205	18.153
C	28.259	60.452	18.461
C	29.027	61.043	19.563
N	29.478	60.251	20.569
N	29.264	62.370	19.534
C	28.812	63.143	18.528
N	28.094	62.668	17.497
C	27.799	61.347	17.421
O	22.714	61.636	16.383
C	23.085	60.383	15.781
C	24.250	60.671	14.847
O	25.183	61.468	15.587
C	24.992	59.441	14.343
O	24.662	59.085	13.007
C	26.407	59.944	14.280
O	26.522	60.388	12.944
C	26.511	61.070	15.307
B	25.567	59.741	12.122

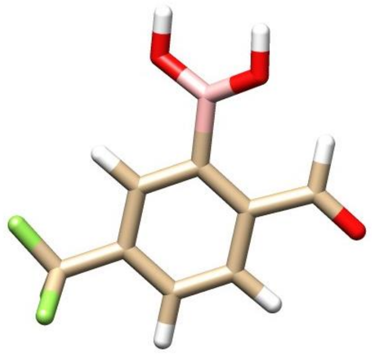

*Fig. S31. Structure of **1***

*Table S10. Coordinates of **1**.*

	Coordinates of the input structure (1) – prior to optimization			Coordinates of the optimized structure (1)
C	-3.69300	-0.80900	-0.16200	1.081 0.414 -0.026
C	-4.30500	-3.56200	-0.21700	-0.906 -1.592 0.004
C	-3.00100	-3.13400	-0.44500	0.431 -1.936 -0.026
C	-2.66400	-1.76500	-0.42500	1.426 -0.952 -0.040
C	-5.00700	-1.27200	0.06600	-0.284 0.739 -0.013
C	-5.32000	-2.64000	0.04100	-1.262 -0.243 0.007
B	-3.49600	0.77900	-0.09600	2.082 1.632 0.013
O	-2.20400	1.43000	-0.27900	3.343 1.471 0.516
C	-6.72400	-3.14000	0.28400	2.840 -1.430 -0.114
C	-1.24000	-1.40500	-0.68600	-2.724 0.114 0.034
O	-4.65100	1.63500	0.17200	1.593 2.827 -0.433
O	-0.40500	-2.27000	-0.90100	3.139 -2.603 -0.096
F	-7.60900	-2.10400	0.52300	-2.934 1.443 0.026
F	-6.72800	-3.98300	1.38100	-3.332 -0.380 1.133
F	-7.16000	-3.84300	-0.82500	-3.378 -0.400 -1.030
H	-0.89500	-0.38800	-0.70000	3.613 -0.652 -0.196
H	-5.00600	1.86600	-0.72400	2.189 3.578 -0.359
H	-1.85000	1.54500	0.63900	3.890 2.263 0.524
H	-4.52400	-4.62300	-0.24100	-0.580 1.778 -0.018
H	-2.25000	-3.88900	-0.64100	-0.906 -1.592 0.004
H	-5.80100	-0.56400	0.26700	0.431 -1.936 -0.026

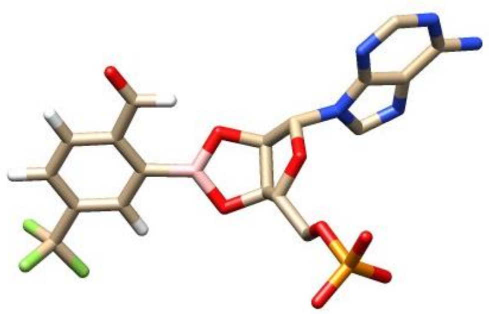

*Fig S32. Structure of the **1-AMP** spiroester*

*Table S11. Coordinates of **1-AMP** spiroester*

P	21.391	61.754	17.311
O	21.558	60.568	18.234
O	20.203	61.660	16.393
O	21.645	63.099	17.972
N	27.150	60.576	16.551
C	27.152	59.299	16.988
N	27.829	59.205	18.153
C	28.259	60.452	18.461
C	29.027	61.043	19.563
N	29.478	60.251	20.569
N	29.264	62.370	19.534
C	28.812	63.143	18.528
N	28.094	62.668	17.497
C	27.799	61.347	17.421
O	22.714	61.636	16.383
C	23.085	60.383	15.781
C	24.250	60.671	14.847
O	25.183	61.468	15.587
C	24.992	59.441	14.343
O	24.662	59.085	13.007
C	26.407	59.944	14.280
C	26.511	61.070	15.307
C	25.509	60.033	10.608
C	25.373	60.145	7.789
C	25.991	61.156	8.498
C	26.067	61.113	9.895
B	25.558	59.817	12.169
C	24.870	59.027	9.867
C	24.806	59.076	8.483
O	26.547	60.398	12.914
C	26.721	62.276	10.567
H	24.419	58.194	10.387
C	24.131	57.985	7.696
H	25.320	60.182	6.709
H	26.429	62.008	7.994
F	23.619	57.019	8.480
F	24.988	57.392	6.839
F	23.116	58.471	6.948
H	26.714	62.270	11.667
O	27.227	63.190	9.955

The best structures obtained in the docking studies to Candida albicans leucyl t-RNA synthetase together with binding energy and the number of structures

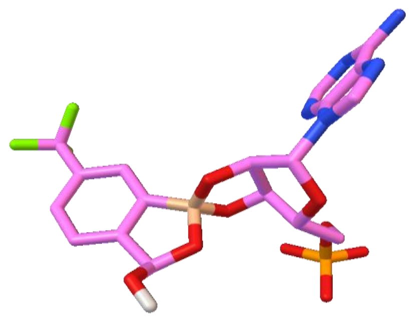

-10.75 kcal/mol (19 structures)

*Fig. S33. The best structure obtained in the docking studies of **1a-S-AMP-structure A** with binding energy and number of structures in the cluster*

*Table S12. Coordinates of the best **1a-S-AMP-structure A** spiroester*

C	27.847	62.257	16.737
C	29.885	63.402	18.244
C	29.404	62.151	18.603
C	28.383	61.599	17.843
B	26.779	61.274	16.171
C	28.333	63.513	16.381
C	29.349	64.079	17.143
O	26.755	60.147	16.966
C	27.707	60.263	18.042
C	24.300	59.651	15.220
O	24.618	58.916	14.032
C	24.839	61.064	15.046
O	25.452	61.587	16.217
C	25.965	60.843	14.075
O	27.081	60.672	14.925
C	25.608	59.589	13.279
O	28.651	59.240	17.999
H	28.218	58.420	18.263
C	29.913	65.431	16.802
F	29.216	66.425	17.397
F	29.906	65.678	15.480
F	31.190	65.558	17.225
C	22.793	59.554	15.402
O	22.321	60.884	15.685
P	21.418	61.185	16.996
O	20.003	61.367	16.520
O	22.127	62.417	17.534
O	21.641	59.934	17.815
N	25.052	59.961	11.955
C	25.094	59.205	10.837
N	24.490	59.848	9.815
C	24.063	61.040	10.297
C	24.447	61.117	11.690
C	23.356	62.196	9.732
N	24.140	62.225	12.408
C	23.480	63.243	11.833
N	23.100	63.243	10.541
N	22.977	62.181	8.428

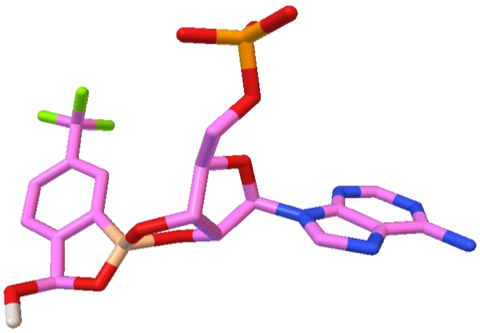

-11.59 kcal/mol (4 structures)

*Fig. S34. The best structure obtained in the docking studies of **1a-S-AMP-structure B** with binding energy and number of structures in the cluster*

*Table S13. Coordinates of the best **1a-S-AMP-structure B** spiroester*

C	24.794	61.290	11.000
C	23.764	62.920	8.998
C	24.450	61.768	8.641
C	24.963	60.971	9.655
B	25.479	60.122	11.772
C	24.105	62.447	11.353
C	23.597	63.258	10.345
O	25.995	59.231	10.852
C	25.739	59.683	9.508
C	24.013	60.591	14.858
O	24.797	61.445	15.701
C	24.955	59.575	14.228
O	24.687	59.327	12.854
C	26.261	60.319	14.247
O	26.293	60.929	12.974
C	26.173	61.317	15.399
O	24.987	58.753	8.795
H	25.397	57.887	8.903
C	22.842	64.518	10.669
F	23.651	65.457	11.212
F	21.836	64.312	11.537
F	22.306	65.075	9.562
C	22.919	59.999	15.733
O	22.753	60.888	16.852
P	21.287	61.435	17.270
O	20.909	60.739	18.549
O	20.496	61.064	16.026
O	21.545	62.917	17.423
N	26.892	60.799	16.588
C	26.965	59.506	16.969
N	27.703	59.391	18.094
C	28.100	60.641	18.435
C	27.554	61.559	17.458
C	28.899	61.216	19.522
N	27.802	62.886	17.577
C	28.552	63.347	18.591
N	29.084	62.552	19.539
N	29.430	60.402	20.470

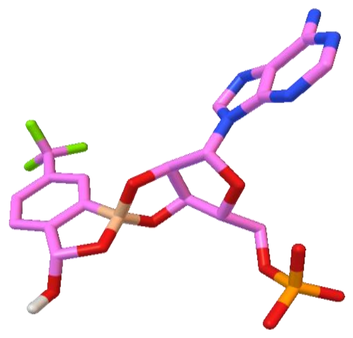

-10.77 kcal/mol (2 structures)

*Fig. S35. The best structure obtained in the docking studies of **1a-R-AMP-structure A** with binding energy and number of structures in the cluster*

*Table S14. Coordinates of the best **1a-R-AMP-structure A** spiroester*

C	25.243	61.187	11.270
C	25.759	60.624	8.598
C	25.053	61.774	8.918
C	24.811	62.042	10.258
B	24.727	61.840	12.587
C	25.952	60.033	10.943
C	26.209	59.762	9.605
O	24.059	63.005	12.267
C	24.072	63.222	10.842
C	24.018	61.046	15.580
O	25.087	61.436	16.451
C	24.635	60.340	14.381
O	24.038	60.695	13.141
C	26.010	60.946	14.345
O	25.852	62.027	13.449
C	26.327	61.381	15.774
O	22.781	63.280	10.324
H	22.612	64.184	10.036
C	26.965	58.529	9.193
F	28.284	58.779	9.046
F	26.848	57.527	10.084
F	26.534	58.056	8.003
C	23.067	60.196	16.408
O	21.792	60.862	16.393
P	21.462	62.052	17.442
O	20.713	63.111	16.680
O	22.867	62.401	17.904
O	20.631	61.326	18.476
N	27.205	60.384	16.434
C	27.567	59.187	15.925
N	28.375	58.537	16.791
C	28.521	59.339	17.872
C	27.742	60.536	17.643
C	29.243	59.243	19.147
N	27.713	61.507	18.588
C	28.407	61.362	19.729
N	29.145	60.271	20.013
N	29.984	58.139	19.418

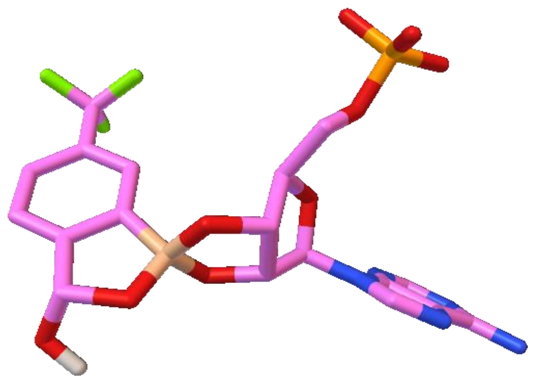

-11.83 kcal/mol (3 structures)

Fig. S36. The best structure obtained in the docking studies of ***1a-R-AMP-structure B*** *with binding energy and number of structures in the cluster*

Table S15. Coordinates of the best ***1a-R-AMP-structure B*** *spiroester*

C	24.603	60.721	11.272
C	23.921	62.209	9.026
C	24.554	60.987	8.855
C	24.881	60.256	9.989
C	23.967	61.949	11.438
C	23.627	62.685	10.309
O	25.711	58.615	11.454
C	25.566	58.911	10.051
C	24.029	60.911	14.897
O	25.028	61.705	15.550
C	24.679	59.595	14.491
O	24.298	59.149	13.196
C	26.122	59.995	14.362
O	26.237	60.314	12.991
C	26.321	61.196	15.285
B	25.224	59.664	12.244
O	26.802	58.976	9.414
H	27.486	58.763	10.060
C	22.943	64.019	10.426
F	22.228	64.115	11.568
F	23.809	65.049	10.413
F	22.070	64.220	9.414
C	22.868	60.783	15.871
O	22.921	61.932	16.735
P	21.653	62.336	17.661
O	22.208	62.932	18.925
O	20.966	60.985	17.780
O	20.929	63.323	16.774
N	26.953	60.769	16.557
C	26.868	59.542	17.112
N	27.566	59.502	18.267
C	28.099	60.734	18.449
C	27.682	61.563	17.339
C	28.940	61.361	19.476
N	28.080	62.858	17.285
C	28.860	63.367	18.253
N	29.278	62.657	19.318
N	29.355	60.631	20.542

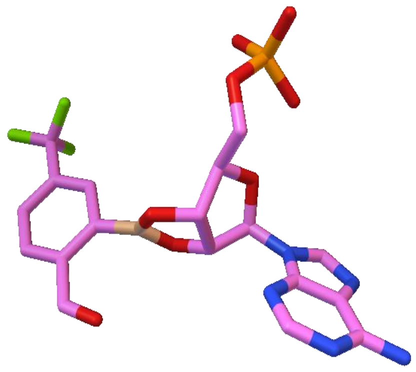

-11.05 kcal/mol (2 structures)

*Fig. S37. The best structure obtained in the docking studies of **1-AMP** with binding energy and number of structures in the cluster*

*Table S16. Coordinates of the best **1-AMP-structure** spiroester*

C	24.730	60.440	15.520
O	25.319	61.575	16.165
C	25.863	59.552	15.023
O	25.638	59.014	13.727
C	26.976	60.547	14.850
C	26.686	61.692	15.820
B	26.152	59.975	12.804
O	26.875	60.933	13.460
C	25.959	60.057	11.241
C	26.693	59.348	10.270
C	26.384	59.480	8.911
C	25.356	60.302	8.493
C	24.630	61.019	9.443
C	24.932	60.901	10.791
C	27.848	58.465	10.612
O	28.242	58.306	11.746
C	23.516	61.915	8.971
F	23.907	63.208	8.930
F	22.429	61.854	9.761
F	23.121	61.595	7.721
C	23.803	59.793	16.536
O	22.489	60.333	16.305
P	22.030	61.726	16.993
O	21.455	62.585	15.900
O	23.350	62.176	17.598
O	21.015	61.248	18.006
N	27.516	61.556	17.041
C	27.884	62.560	17.865
N	28.635	62.079	18.880
C	28.747	60.743	18.689
C	28.029	60.408	17.478
C	29.413	59.645	19.399
N	27.994	59.120	17.057
C	28.616	58.160	17.760
N	29.309	58.401	18.890
N	30.106	59.903	20.538

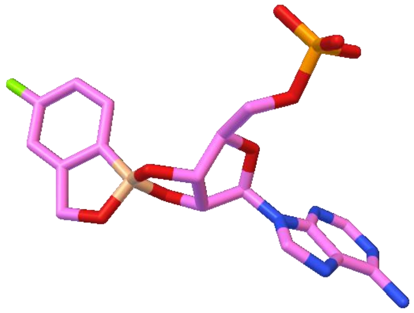

-11.89 kcal/mol (17 structures)

Fig. S38. The best structure obtained in the docking studies of ***AN2690-AMP*** *with binding energy and number of structures in the cluster*

Table S17. Coordinates of the best ***AN2690-AMP*** *spiroester*

C 26.648 58.662 9.988

C 25.666 59.760 9.650

C 27.089 59.235 17.052

N 27.773 59.163 18.215

C 28.282 60.396 18.450

C 29.098 60.998 19.510

C 27.865 61.259 17.366

N 29.512 60.235 20.555

N 29.415 62.304 19.406

C 28.999 63.047 18.363

C 25.293 60.256 8.406

C 25.085 60.284 10.875

C 24.348 61.279 8.343

C 24.129 61.313 10.796

C 23.770 61.813 9.522

C 24.289 60.512 14.790

O 25.199 61.334 15.531

C 25.055 59.283 14.321

O 24.753 58.899 12.986

C 26.465 59.802 14.274

O 26.598 60.223 12.932

C 26.537 60.947 15.282

F 24.000 61.740 7.124

O 26.477 58.524 11.417

B 25.665 59.550 12.105

N 27.160 60.483 16.545

N 28.241 62.562 17.366

C 23.112 60.227 15.708

O 23.163 61.200 16.767

P 21.811 61.904 17.317

O 21.120 60.746 18.000

O 21.078 62.440 16.117

O 22.409 62.925 18.271

The best structures obtained in the docking studies to *Escherichia coli* leucyl t-RNA synthetase together with binding energy and the number of structures

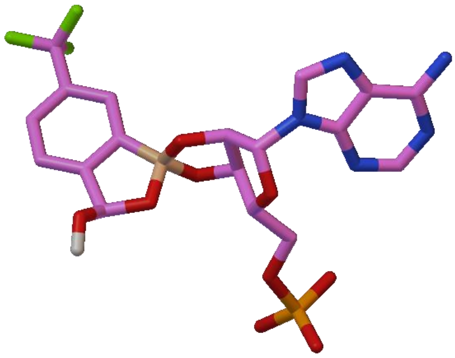

-7.54 kcal/mol (12 structures)

*Fig. S39. The best structure obtained in the docking studies of **1a-S-AMP-structure A** with binding energy and number of structures in the cluster*

*Table S18. Coordinates of the best **1a-S-AMP-structure A** spiroester*

C	28.328	26.883	31.760
C	30.831	25.750	32.187
C	30.646	27.097	32.464
C	29.392	27.645	32.239
B	27.127	27.873	31.700
C	28.519	25.532	31.482
C	29.775	24.975	31.695
O	27.559	29.113	32.123
C	28.959	29.075	32.465
C	24.784	29.821	31.065
O	23.776	29.726	32.079
C	25.188	28.404	30.682
O	26.589	28.241	30.501
C	24.862	27.645	31.938
O	26.089	27.656	32.638
C	23.753	28.427	32.640
O	29.168	29.430	33.795
H	29.394	30.368	33.823
C	30.038	23.521	31.415
F	29.013	22.741	31.829
F	31.150	23.068	32.021
F	30.182	23.287	30.093
C	24.190	30.648	29.936
O	25.229	31.530	29.474
P	25.026	32.430	28.142
O	24.444	33.744	28.587
O	24.121	31.515	27.334
O	26.441	32.523	27.618
N	22.435	27.800	32.378
C	21.890	26.779	33.073
N	20.679	26.462	32.566
C	20.457	27.298	31.524
C	21.613	28.159	31.394
C	19.369	27.487	30.557
N	21.644	29.093	30.411
C	20.609	29.221	29.566
N	19.507	28.449	29.622
N	18.263	26.702	30.628

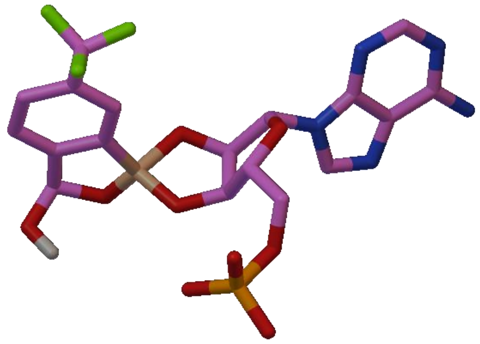

-7.72 kcal/mol (4 structures)

*Fig. S40. The best structure obtained in the docking studies of **1a-S-AMP-structure B** with binding energy and number of structures in the cluster*

*Table S19. Coordinates of the best **1a-S-AMP-structure B** spiroester*

C	24.347	31.894	30.468
C	23.405	34.450	31.023
C	23.191	33.914	29.762
C	23.675	32.639	29.502
B	24.666	30.538	29.772
C	24.560	32.437	31.733
C	24.088	33.718	32.000
O	24.181	30.584	28.480
C	23.563	31.858	28.214
C	25.287	28.630	32.577
O	26.675	28.633	32.932
C	25.196	28.388	31.077
O	24.221	29.195	30.429
C	26.522	28.913	30.603
O	26.226	30.251	30.263
C	27.484	28.782	31.782
O	22.224	31.709	27.862
H	21.948	30.816	28.100
C	24.287	34.352	33.349
F	23.187	34.225	34.126
F	24.565	35.666	33.268
F	25.300	33.772	34.027
C	24.618	27.574	33.442
O	23.784	26.789	32.571
P	22.183	27.029	32.511
O	21.900	27.773	31.234
O	21.977	27.790	33.810
O	21.659	25.611	32.533
N	28.337	27.579	31.621
C	28.066	26.514	30.838
N	29.059	25.603	30.927
C	29.975	26.102	31.790
C	29.492	27.385	32.254
C	31.259	25.635	32.327
N	30.230	28.096	33.141
C	31.403	27.616	33.585
N	31.909	26.428	33.203
N	31.757	24.436	31.932

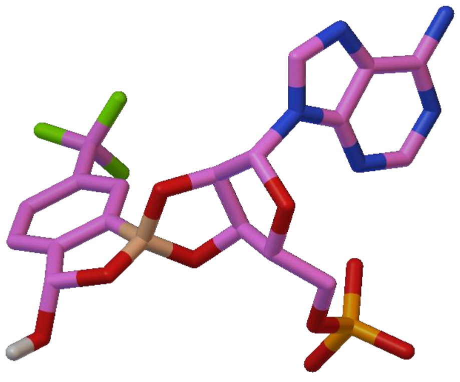

-7.54 kcal/mol (21 structures)

*Fig. S41. The best structure obtained in the docking studies of **1a-R-AMP-structure A** with binding energy and number of structures in the cluster*

*Table S20. Coordinates of the best **1a-R-AMP-structure A** spiroester*

C	11.632	37.797	26.802
C	12.457	39.752	28.597
C	12.912	39.808	27.288
C	12.484	38.827	26.405
B	11.451	36.919	25.528
C	11.179	37.747	28.118
C	11.592	38.730	29.008
O	12.178	37.484	24.499
C	12.842	38.683	24.945
C	10.660	33.953	24.712
O	9.301	33.921	24.259
C	10.697	34.756	26.005
O	11.808	35.637	26.097
C	9.498	35.648	25.843
O	10.050	36.814	25.268
C	8.533	34.910	24.916
O	14.223	38.584	24.800
H	14.485	39.117	24.041
C	11.138	38.723	30.442
F	11.845	37.844	31.185
F	11.260	39.927	31.029
F	9.842	38.356	30.550
C	11.112	32.507	24.839
O	12.403	32.527	25.475
P	12.670	31.701	26.843
O	11.492	31.953	27.743
O	14.000	32.302	27.265
O	12.766	30.282	26.329
N	7.459	34.257	25.704
C	6.153	34.597	25.699
N	5.457	33.791	26.529
C	6.345	32.926	27.075
C	7.654	33.244	26.547
C	6.261	31.821	28.038
N	8.730	32.522	26.945
C	8.582	31.515	27.821
N	7.397	31.169	28.359
N	5.059	31.483	28.570

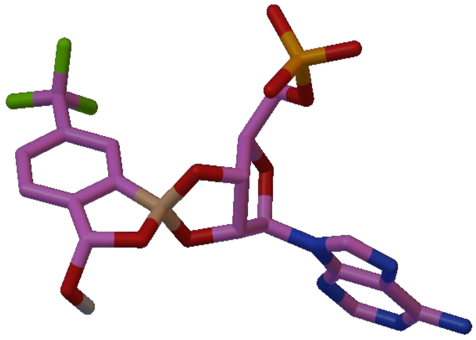

-8.15 kcal/mol (2 structures)

*Fig. S42. The best structure obtained in the docking studies of **1a-R-AMP-structure B** with binding energy and number of structures in the cluster*

*Table S21. Coordinates of the best **1a-R-AMP-structure B** spiroester*

C	24.575	32.050	30.808
C	24.087	34.712	31.439
C	23.993	34.307	30.116
C	24.234	32.972	29.823
C	24.668	32.462	32.136
C	24.419	33.794	32.443
O	24.559	30.932	28.700
C	24.190	32.305	28.467
C	25.184	28.922	32.640
O	26.575	28.744	32.935
C	24.994	28.682	31.149
O	24.108	29.605	30.531
C	26.356	29.021	30.610
O	26.225	30.385	30.265
C	27.344	28.774	31.749
B	24.864	30.713	30.049
O	25.080	32.932	27.599
H	25.975	32.671	27.846
C	24.508	34.297	33.857
F	24.633	35.641	33.902
F	23.427	33.973	34.590
F	25.587	33.794	34.496
C	24.420	27.971	33.548
O	23.946	26.894	32.719
P	22.360	26.634	32.519
O	22.010	27.109	31.135
O	21.806	27.439	33.683
O	22.270	25.136	32.702
N	28.023	27.468	31.568
C	27.463	26.346	31.068
N	28.368	25.344	31.042
C	29.524	25.843	31.541
C	29.289	27.225	31.900
C	30.865	25.304	31.792
N	30.297	27.958	32.434
C	31.506	27.406	32.627
N	31.792	26.125	32.326
N	31.139	24.011	31.481

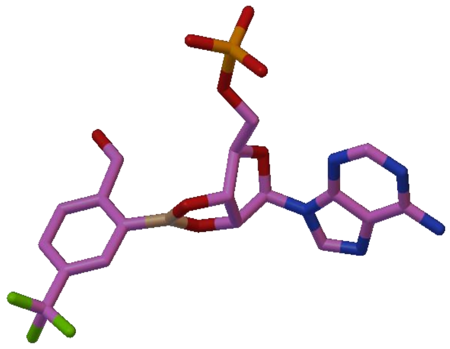

-8.23 kcal/mol (4 structures)

Fig. S43. The best structure obtained in the docking studies of ***1-AMP*** *with binding energy and number of structures in the cluster*

Table S22. Coordinates of the best ***1-AMP*** *spiroester*

C	25.043	29.558	31.048
O	23.993	29.587	32.024
C	25.379	28.098	30.777
O	26.774	27.847	30.664
C	24.963	27.445	32.065
C	23.875	28.333	32.667
B	27.237	27.580	31.989
O	26.181	27.421	32.844
C	28.716	27.489	32.527
C	29.358	28.419	33.368
C	30.666	28.190	33.812
C	31.352	27.051	33.440
C	30.732	26.127	32.598
C	29.440	26.348	32.148
C	28.721	29.701	33.796
O	29.318	30.564	34.398
C	31.496	24.894	32.198
F	30.763	24.102	31.384
F	31.865	24.145	33.253
F	32.618	25.207	31.518
C	24.540	30.338	29.843
O	25.540	31.327	29.541
P	25.323	32.389	28.337
O	24.665	33.602	28.936
O	24.483	31.551	27.386
O	26.743	32.619	27.871
N	22.534	27.763	32.393
C	21.946	26.756	33.073
N	20.725	26.494	32.558
C	20.541	27.351	31.525
C	21.731	28.166	31.410
C	19.466	27.595	30.557
N	21.805	29.109	30.438
C	20.779	29.289	29.591
N	19.646	28.561	29.634
N	18.329	26.855	30.614

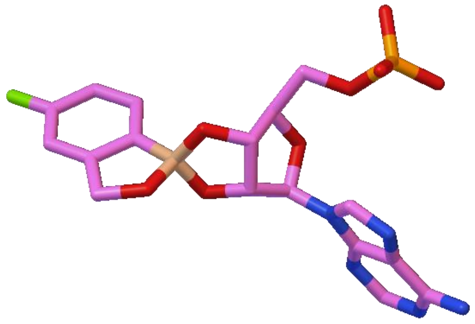

-8.27 kcal/mol (22 structures)

*Fig. S44. The best structure obtained in the docking studies of **AN2690-AMP** with binding energy and number of structures in the cluster*

*Table S23. Coordinates of the best **AN2690-AMP** spiroester*

C	30.990	28.096	34.155
C	31.010	26.823	33.341
C	32.046	25.916	33.151
C	29.706	26.617	32.732
C	31.822	24.793	32.355
C	29.493	25.477	31.935
C	30.563	24.570	31.744
C	25.830	27.265	31.980
O	25.581	28.152	30.883
C	26.452	28.083	33.103
O	27.522	27.421	33.765
C	27.095	29.207	32.340
O	28.412	28.738	32.142
C	26.301	29.359	31.044
F	32.841	23.926	32.190
O	29.603	28.495	34.071
B	28.743	27.764	33.115
N	25.348	30.490	31.151
C	24.548	30.755	32.206
N	23.813	31.863	31.968
C	24.152	32.303	30.733
C	23.739	33.424	29.880
C	25.142	31.394	30.196
N	22.809	34.304	30.330
N	24.310	33.544	28.665
C	25.234	32.664	28.232
N	25.647	31.611	28.957
C	24.502	26.608	32.321
O	23.468	27.478	31.827
P	21.946	27.353	32.369
O	21.692	25.869	32.230
O	21.092	28.212	31.476
O	22.131	27.823	33.802

9. Pictures of chosen results of diffusion agar method

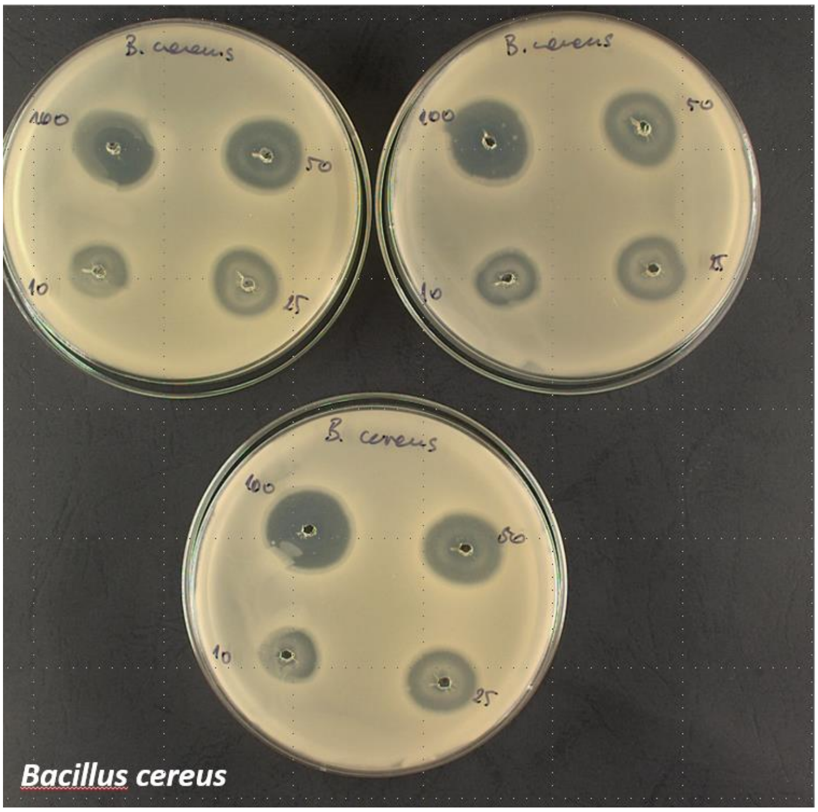

Fig. S45. Results of the agar diffusion method of activity of **1** against *Bacillus cereus* in triplicate

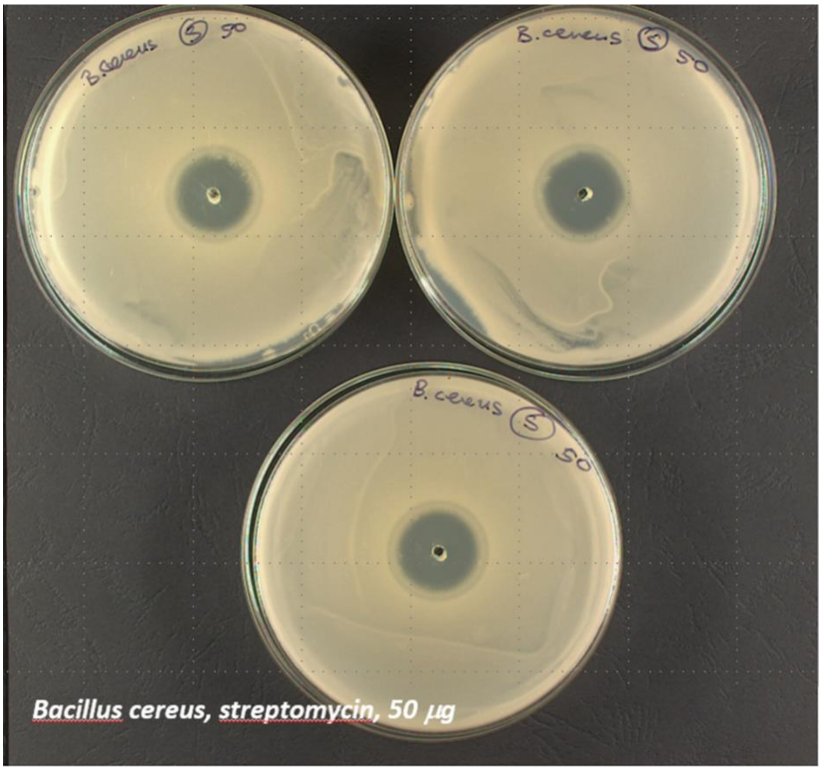

Fig. S46. Results of the agar diffusion method of activity of **streptomycin** at 50μg against *Bacillus cereus* in triplicate

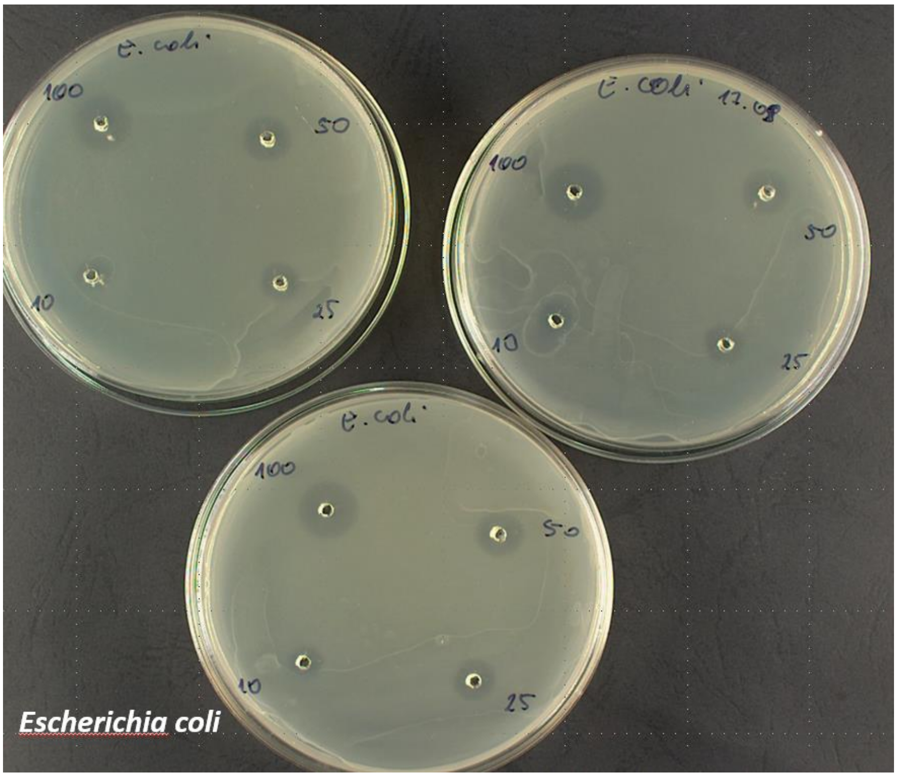

Fig. S47. Results of the agar diffusion method of activity of **1** against *Escherichia coli* in triplicate

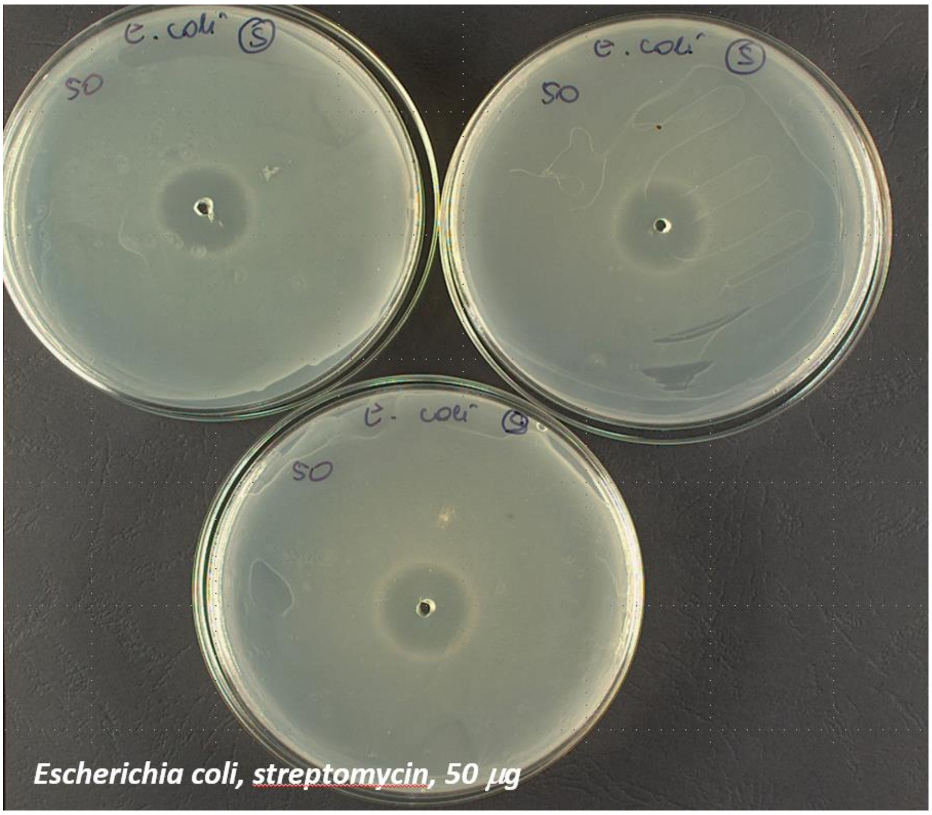

Fig. S48. Results of the agar diffusion method of activity of **streptomycin** at 50µgagainst *Escherichia coli* in triplicate

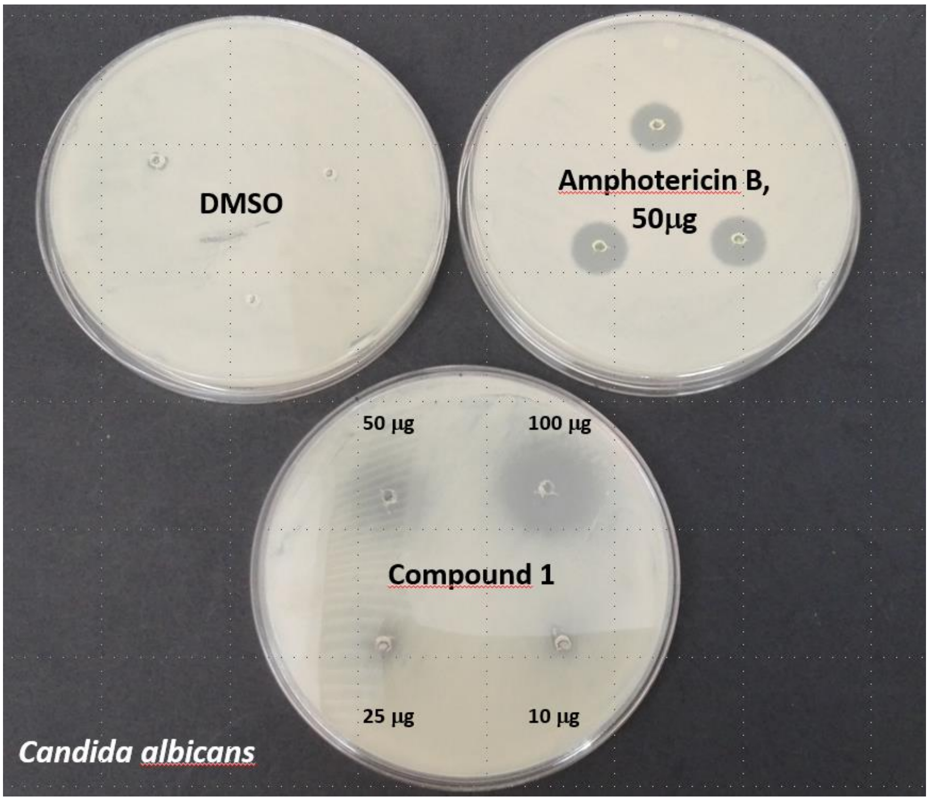

Fig. S49. Results of the agar diffusion method of activity of **1** (10, 25, 50 and 100μg), DMSO and Amphotericin B (50μg) against *Candida albicans*

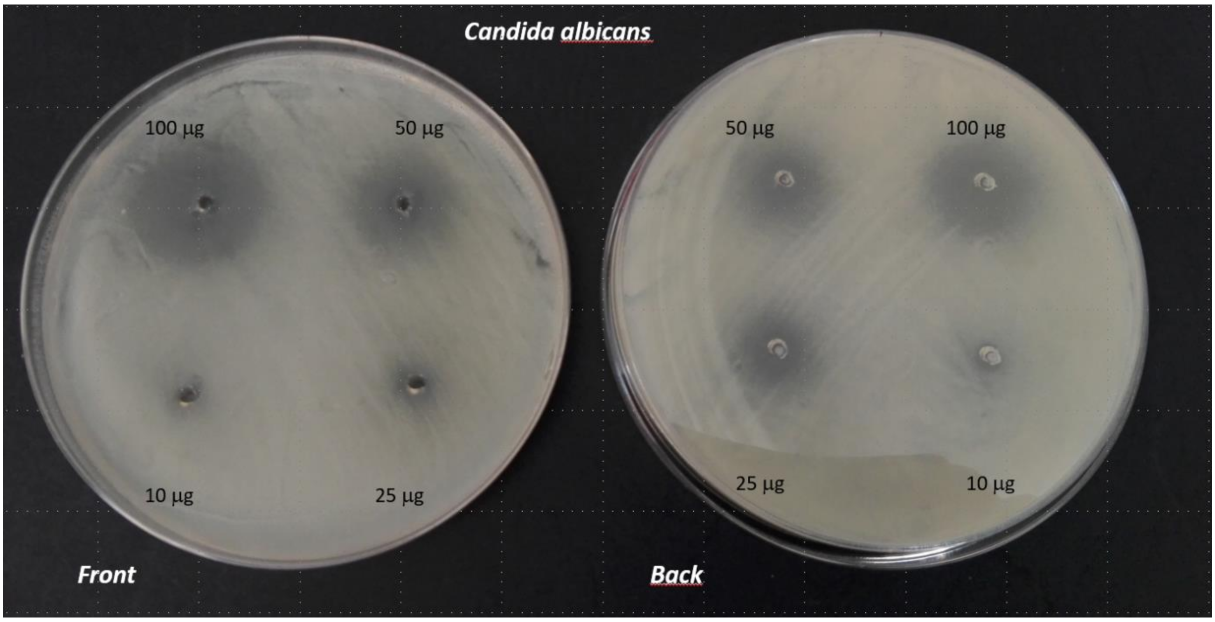

Fig. S50. Results of the agar diffusion method of activity of **1** (10, 25, 50 and 100μg) against *Candida albicans* (front and back).

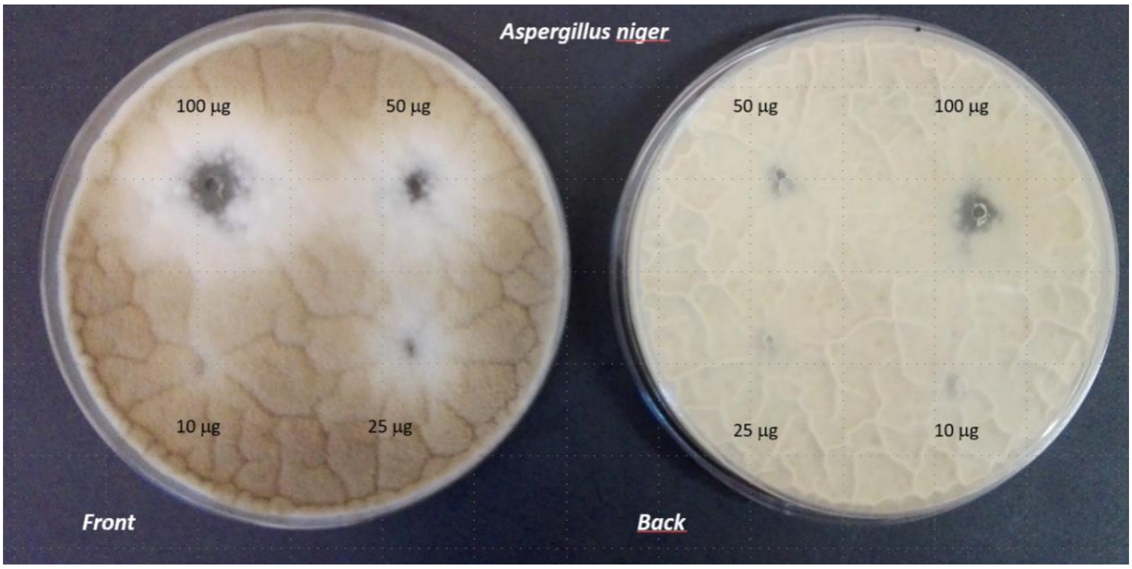

Fig. S51. Results of the agar diffusion method of activity of **1** (10, 25, 50 and 100µg) against *Aspergillus niger* (front and back).

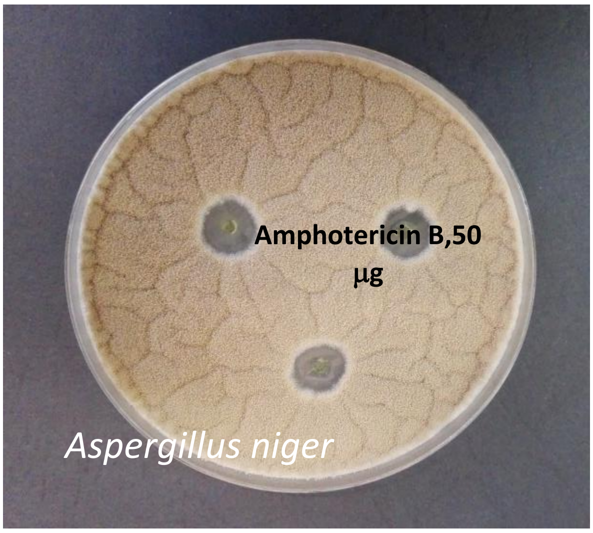

Fig. S52. Results of the agar diffusion method of activity of Amphotericin B (50 µg) against *Aspergillus niger.*
